# Depot medroxyprogesterone acetate (DMPA) enhances susceptibility and increases the window of vulnerability to HIV-1 in humanized mice

**DOI:** 10.1038/s41598-021-83242-9

**Published:** 2021-02-16

**Authors:** Jocelyn M. Wessels, Philip V. Nguyen, Danielle Vitali, Kristen Mueller, Fatemeh Vahedi, Allison M. Felker, Haley A. Dupont, Puja Bagri, Chris P. Verschoor, Alexandre Deshiere, Tony Mazzulli, Michel J. Tremblay, Ali A. Ashkar, Charu Kaushic

**Affiliations:** 1 McMaster Immunology Research Centre, Michael G. DeGroote Centre for Learning and Discovery, McMaster University, MDCL Room 4014, 1280 Main Street West, Hamilton, ON L8S 4K1 Canada; 2 Department of Pathology and Molecular Medicine, McMaster University, 1280 Main Street West, Hamilton, ON L8S 4K1 Canada; 3 Axe Des Maladies Infectieuses Et Immunitaires, Centre de Recherche du CHU de Québec-Université Laval, Pavillon CHUL, Québec City, QC G1V 4G2 Canada; 4 Public Health Laboratories, Public Health Ontario, Toronto, ON M5G 1V2 Canada; 5 Department of Microbiology, Mount Sinai Hospital/University Health Network, Toronto, ON M5G 1X5 Canada; 6 Department of Laboratory Medicine and Pathobiology, University of Toronto, Toronto, ON M5S 1A8 Canada

**Keywords:** Immunology, Infectious diseases, HIV infections

## Abstract

The progestin-based hormonal contraceptive Depot Medroxyprogesterone Acetate (DMPA) is widely used in sub-Saharan Africa, where HIV-1 is endemic. Meta-analyses have shown that women using DMPA are 40% more likely than women not using hormonal contraceptives to acquire Human Immunodeficiency Virus (HIV-1). Therefore understanding how DMPA increases susceptibility to HIV-1 is an important public health issue. Using C57BL/6 mice and our previously optimized humanized mouse model (*NOD-Rag1*^*tm1Mom*^* Il2rg*^*tm1Wjl*^ transplanted with hCD34-enriched hematopoietic stem cells; Hu-mice) where peripheral blood and tissues are reconstituted by human immune cells, we assessed how DMPA affected mucosal barrier function, HIV-1 susceptibility, viral titres, and target cells compared to mice in the diestrus phase of the estrous cycle, when endogenous progesterone is highest. We found that DMPA enhanced FITC-dextran dye leakage from the vaginal tract into the systemic circulation, enhanced target cells (hCD68+ macrophages, hCD4+ T cells) in the vaginal tract and peripheral blood (hCD45+hCD3+hCD4+hCCR5+ T cells), increased the rate of intravaginal HIV-1 infection, extended the window of vulnerability, and lowered vaginal viral titres following infection. These findings suggest DMPA may enhance susceptibility to HIV-1 in Hu-mice by impairing the vaginal epithelial barrier, increasing vaginal target cells (including macrophages), and extending the period of time during which Hu-mice are susceptible to infection; mechanisms that might also affect HIV-1 susceptibility in women.

## Introduction

Many sexually transmitted infections (STIs) are more common in women than men^1^, including human immunodeficiency virus (HIV-1) where more than half of infections occur in women^2^. There are greater than 36.7 M people living with HIV world-wide, and 1.8 M new infections still occur each year^2^. It has been estimated that approximately 40% of all HIV-1 infections originate in the female genital tract^3,4^. A number of socio-economic and biological factors are known to contribute to HIV-1 acquisition in women, including the hormonal status of women and the stage of the menstrual cycle. Previous studies have shown that in primate models^5–8^ as well as in ex vivo explant cultures^9^, higher HIV infection occurred in the progesterone high phase of the cycle. Related to this, hormonal contraceptives, especially Depot Medroxyprogesterone Acetate (DMPA) have come under close scrutiny to examine their effect on HIV-1 susceptibility. DMPA is an injectable, progestin-based hormonal contraceptive that is associated in meta-analyses with a 40–50% increased risk of HIV-1 compared to women not taking hormonal contraception^10,11^. It is a common contraceptive in sub-Saharan Africa where it is used by over 8 M women^12^. As such, DMPA is a public health concern because this geographical region also has the greatest prevalence of HIV-1^2^.

Several mechanisms linking hormonal contraceptives including DMPA to increased HIV-1 susceptibility have been described and are reviewed elsewhere^13^. Briefly, these mechanisms include modification of the protective epithelial barrier lining the female genital tract, altered genital immunity and defense (cytokines, chemokines, defensins, antibodies, etc.), a change in HIV-1 target cell activation or frequency (T cells, dendritic cells (DCs), and macrophages), and most recently modification of the vaginal microbiota^13,14^. We recently characterized and described an intravaginal infection model of HIV-1 in humanized mice^15^ (Hu-mice) where we demonstrated that frequency of human CD45+ target cells was the primary determinant of HIV-1 infection following intravaginal inoculation, and that viral dose was a determinant of viral dissemination and plasma titres during early infection^15^. We also demonstrated the classical loss of hCD4+ cells in our Hu-mice following intravaginal HIV-1 infection. We subsequently found that DMPA enhanced the rate of HIV-1 acquisition in this Hu-mouse model^14^. However we did not examine the detailed mechanism(s) by which DMPA was enhancing HIV-1 infection. Herein, we used our previously well-described Hu-mice^14,15^ and C57BL/6 mice to experimentally evaluate the effects of DMPA on HIV-1 target cells and susceptibility, and to probe the mechanisms by which it elicits increased susceptibility. We found that DMPA impaired the integrity of the murine vaginal epithelial barrier, and significantly increased the number of Hu-mice that became infected following intravaginal inoculation with HIV-1, as compared to humanized mice challenged during diestrus, the progesterone-high phase of the estrous cycle. We also found that the window of susceptibility following viral challenge was extended in DMPA-treated humanized mice, indicating that DMPA not only increases the likelihood of HIV-1 infection but also prolongs the time during which humanized mice are vulnerable to infection. Following HIV-1 infection, viral titres were lower in the vaginal washes of DMPA-treated humanized mice during early infection, while titres in blood were similar. Viral dissemination was initially slowed by treatment with DMPA, but by 5 weeks post-infection was found to be similar to Hu-mice infected at diestrus. Although there were no major differences in vaginal cytokines in uninfected (never exposed) humanized mice, DMPA-treated humanized mice had an enhanced macrophage population by 1 week post-DMPA, and enhanced T cells in the vagina and peripheral blood at 4 weeks post-DMPA. Taken together, our results demonstrate that DMPA may enhance susceptibility to HIV-1 in Hu-mice by increasing vaginal target cells and lengthening the window of viral susceptibility likely by increasing target cells in blood and the vaginal tract.

## Results

### DMPA impairs integrity of the murine vaginal epithelial barrier

**Figure 1:**
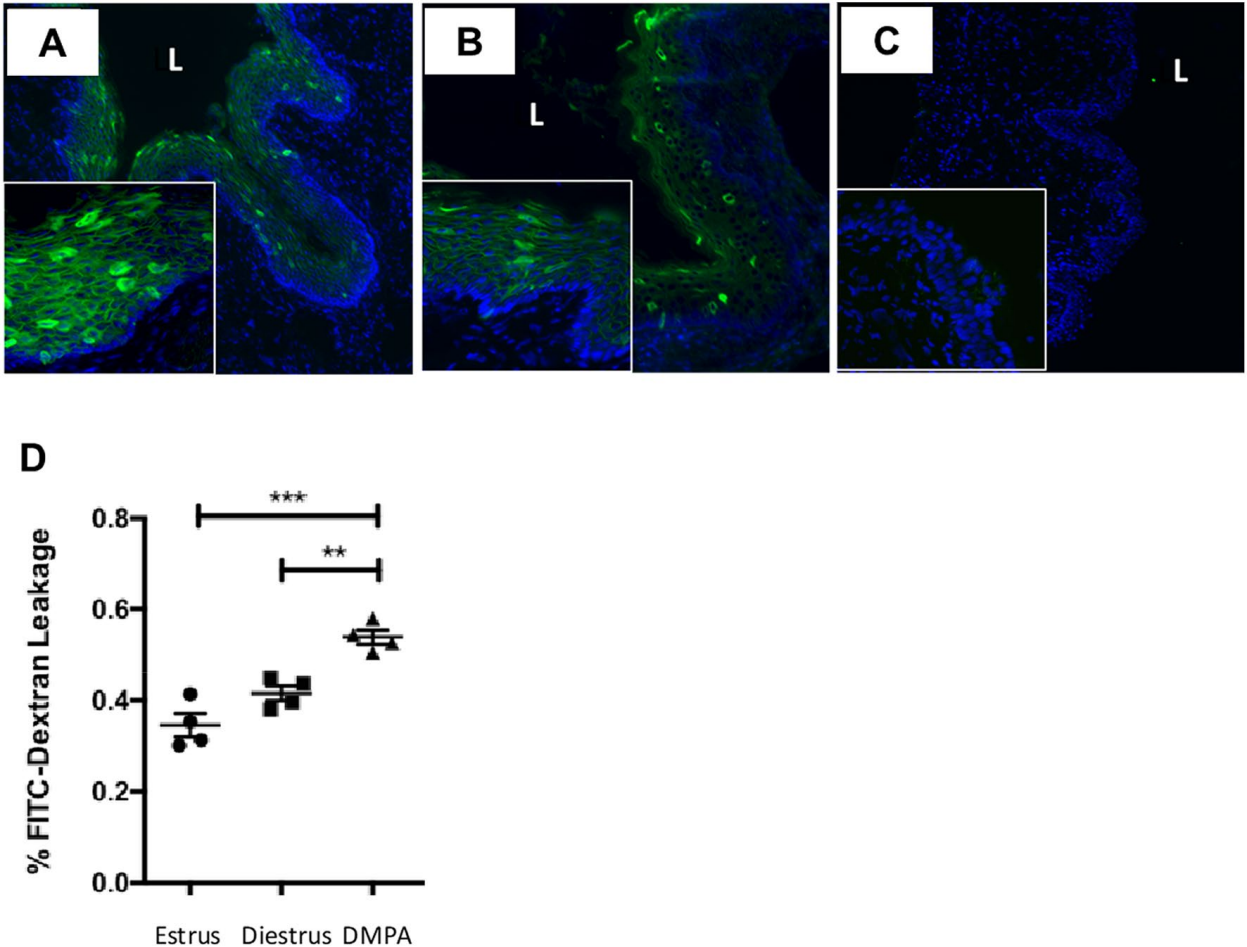
DMPA impairs integrity of the murine vaginal epithelial barrier. C57BL/6 mice were sacrificed at estrus (N = 4) (**A**), diestrus (N = 4) (**B**), or 1 week following a subcutaneous injection of 2 mg of DMPA (N = 4) (**C**). Vaginal tissues were stained by immunofluorescence for the cell–cell adhesion molecule desmoglein-1 (green), and counterstained with DAPI (blue). Mice treated with DMPA appeared to have less desmoglein-1α staining as compared to those in estrus or diestrus. (**D**) We also assessed the murine vaginal barrier in a separate cohort of mice (N = 4/group) by administering a 4 kDa fluorescein isothiocyanate-dextran (FITC-dextran) dye into the vaginal lumen and measuring the fluorescence within the peripheral circulation at sacrifice. Significantly more FITC-dextran dye was quantified in the serum of mice that had received DMPA than those sacrificed during estrus or diestrus. (One-way ANOVA). Taken together DMPA negatively impacts vaginal cell-adhesion molecules, and enhances leakage from the vaginal lumen into the submucosa, ultimately impairing the integrity of the murine vaginal epithelial barrier. **P ≤ 0.01. ***P ≤ 0.001. Magnifications: 100× and inset is 400×. Data in D is presented as mean ± SEM. *DMPA* Depot-medroxyprogesterone acetate, *L* vaginal lumen.

The vaginal epithelial barrier provides the first line of defense against STIs like HIV-1. We therefore first sought to determine if DMPA negatively impacted the vaginal epithelial barrier in C57BL/6 mice, as has been reported in primates^16–18^, and another study in mice^19^, which could potentially enhance viral infection^20^. Our experimental designs are presented in Supplemental Fig. 1. In our first experiment C57BL/6 mice treated with DMPA were found to have decreased immunofluorescent staining for the cell–cell adhesion molecule desmoglein-1α in the vaginal epithelium as compared to mice in estrus (high endogenous estrogen) or diestrus (high endogenous progesterone) (N = 4/group) (Fig. 1A–C). We were also able to assess the integrity of the murine vaginal barrier in a separate group of C57BL/6 mice by administering fluorescein isothiocyanate-dextran (FITC-dextran) dye directly into the vaginal lumen and measuring the fluorescence in blood samples from mice 4 h later; an indicator of how much dye leaked across the vaginal barrier into peripheral circulation. Using this assay, the vaginal barrier function was compared at estrus, diestrus, 1 week following treatment with DMPA (N = 4/group) to determine if DMPA enhanced leakage of the dye into the peripheral circulation, above what could be observed during the estrogen-high phase of the murine estrous cycle (estrus), and the progesterone-high phase of the estrous cycle (diestrus). Significantly more FITC-dextran dye was quantified in the serum of mice that had received DMPA than those sacrificed during estrus or diestrus (Fig. 1D). Taken together, these two experiments demonstrate that DMPA negatively impacts vaginal cell-adhesion molecules and enhances leakage from the vaginal lumen into the submucosa, ultimately impairing the integrity of the murine vaginal epithelial barrier.

### DMPA increases HIV-1 infection in humanized mice

In addition to its effect on the vaginal barrier, DMPA may enhance viral infection through other mechanisms^13^. In our Hu-mouse model, as in non-human primates (NHP)^21–23^, and women^10,11^, we observed increased HIV-1 acquisition following intravaginal exposure^14^. Additionally, our previous work^15^ found that target cells and inoculation dose are determinants of HIV-1 susceptibility. Herein, we thus set out to see if we could determine how DMPA affects intravaginal HIV-1 infection in this Hu-mouse model. Our experimental designs are presented in Supplemental Fig. 1.

**Figure 2:**
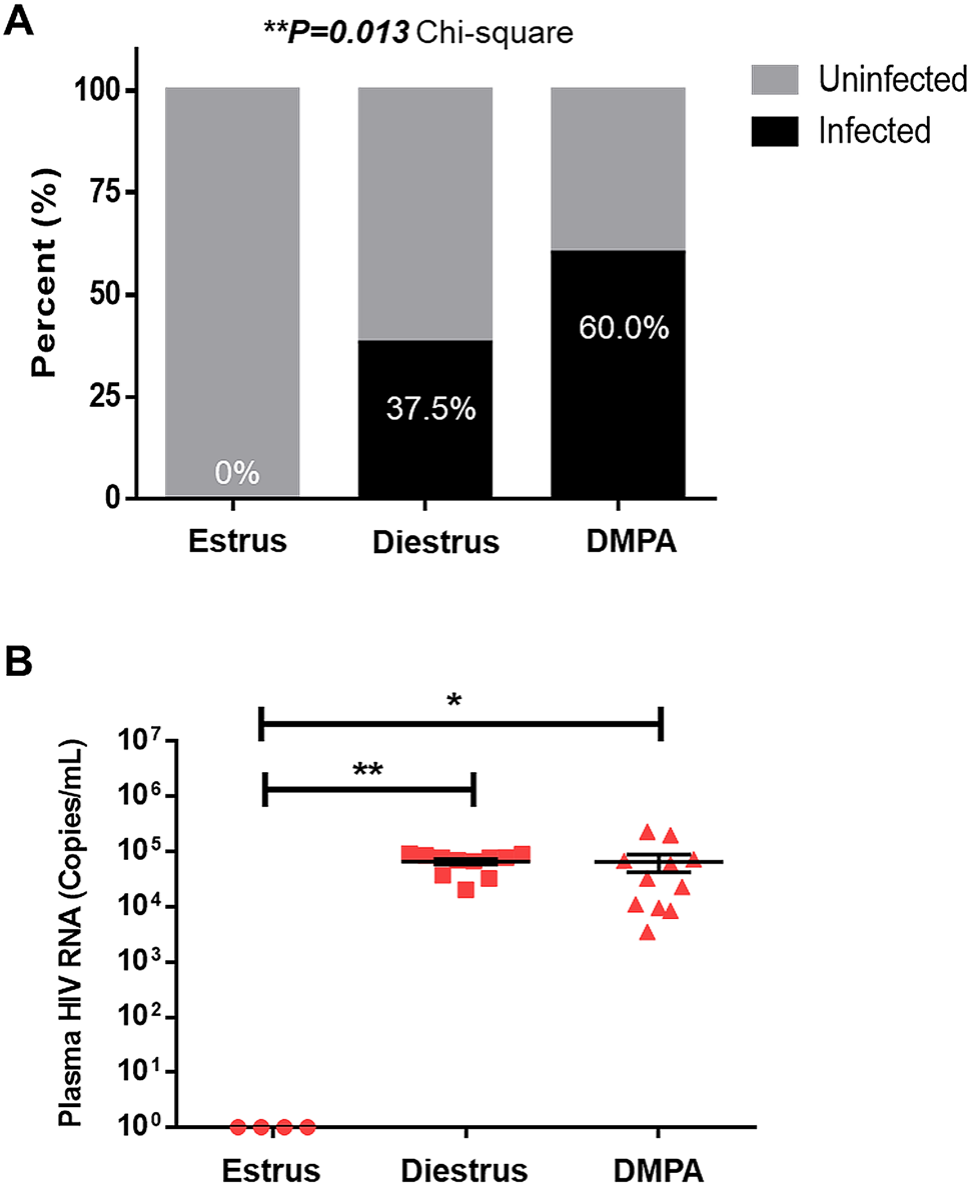
DMPA increases HIV-1 infection in humanized mice. (**A**) Hu-mice were intravaginally challenged with HIV-1 during estrus (N = 4), diestrus (N = 40), or 1 week after receiving DMPA (N = 55). Significantly more Hu-mice treated with DMPA became infected with HIV-1 (33/55 (60.0%)) than those challenged during estrus (0/4 (0%)) or diestrus (15/40 (37.5%)) (*P* = 0.013, Chi-square). (**B**) HIV-1 titres were quantified in the blood of Hu-mice 3 weeks after intravaginal challenge. Hu-mice challenged at estrus (N = 4) did not become infected and had undetectable viral titres compared with those challenged at diestrus (N = 11) or following DMPA (N = 11; *P* = 0.0029, Kruskal–Wallis test by ranks). *P ≤ 0.05. **P ≤ 0.01. Data in (**A**) is presented as proportions (percent infected), data in (**B**) is presented as mean ± SEM. *DMPA* Depot-medroxyprogesterone acetate, *IVAG* intravaginally.

Briefly, Hu-mice were reconstituted with hCD34 hematopoietic stem cells as described in Methods and “humanization” (%hCD45+ cells in peripheral blood) was assessed 90–120 days later by flow cytometry (Supplemental Fig. 2; Supplemental File 1). Because our previous work^15^ found that target cells were the primary determinant of HIV-1 susceptibility in these Hu-mice, Hu-mice with > 10% hCD45+ cells in peripheral blood and < 10% hCD45 hCD45+ cells in peripheral blood were evenly distributed between our experimental groups (DMPA and diestrus), and intravaginally challenged with the HIV-1 R5 strain (NLR4.3-Bal-Env). Reconstitution levels (“humanization” levels, %hCD45 in peripheral blood 90–120 days following intrahepatic injection of CD34 enriched hematopoietic stem cells) of the DMPA and diestrus Hu-mice can be found in Supplemental File 1. The overall proportion of HIV-1 infection following intravaginal exposure in Hu-mice was 0/4 (0%) when Hu-mice were challenged during the estrus (estrogen high) phase of the estrous cycle, 15/40 (37.5%) when challenged during the diestrus (progesterone high) phase of the estrous cycle, and 33/55 (60.0%) when Hu-mice were challenged 1 week after receiving a subcutaneous dose of 2 mg of DMPA (Fig. 2A). The number of infections was significantly affected by cycle/hormonal status (*P* = 0.013, Chi-square). Plasma viral titres were compared in Hu-mice that had been intravaginally challenged with HIV-1 during estrus (N = 4), diestrus (N = 11), and DMPA (N = 11), 3 weeks following exposure (Fig. 2B). Hu-mice challenged at estrus did not become infected and had no detectable viral titers. Hu-mice infected at diestrus or following administration of DMPA did not show any significant differences in viral shedding (6.6 × 10^4^ ± 7.5 × 10^3^ vs. 6.5 × 10^4^ ± 2.3 × 10^4^ HIV-1 copies/mL respectively; *P* = 0.12, Mann–Whitney). From this we concluded that DMPA treated Hu-mice have increased susceptibility to intravaginal (IVAG) HIV-1, but similar levels of viral replication in peripheral blood as Hu-mice infected during diestrus, at 3 weeks post-infection.

In order to confirm that DMPA increased the odds of infection following IVAG exposure to HIV-1, the association between HIV-1 infection in Hu-mice following HIV-1 exposure and covariables was estimated using a univariate (unadjusted) and multivariate (adjusted) generalized linear model (Table 1). In the univariate analysis Hu-mice treated with DMPA (OR 2.50, 95% CI 1.09–5.88; *P* = 0.032), or with > 10% circulating hCD45+ cells (OR 2.92, 95% CI 1.25–7.11; *P* = 0.015), or challenged with high viral dose (10^5^) (OR 2.94, 95% CI 1.29–6.89; *P* = 0.011) were more likely to become infected following IVAG HIV-1 challenge. After adjusting for CD45 and viral dose, DMPA-treated Hu-mice were 3.25 times more likely to become infected with HIV-1 following IVAG exposure than those inoculated during diestrus (3.25 aOR, 1.28–8.86 CI; *P* = 0.016; N = 95; 55 DMPA, 40 diestrus) (Table 1). The frequency of hCD45+ target cells in the plasma as well as the viral inoculation dose had already been demonstrated as a determinant of HIV-1 infection in our previous study (*P* = 0.01537)^15^. To ensure that peripheral blood target cell frequency was equivalent between the Hu-mice challenged during diestrus and those challenged post-DMPA the average reconstitution (circulating hCD45+ cells) was statistically compared between groups and was not found to be significantly different (10.8 ± 1.7% DMPA versus 12.5 ± 2.1% diestrus; *P* = 0.5223 Mann–Whitney). Furthermore, we observed the classical loss of HIV-1 target cells (hCD45+hCD3+) over time in our infected Hu-mice. This was observed in Hu-mice regardless of whether the Hu-mice had or had not received DMPA, and in those that received a high (10^5^) or low (10^3^) viral dose at challenge (Supplemental File 2). This decrease in frequency of hCD45+hCD3+ cells was not seen in Hu-mice that did not become infected following intravaginal HIV-1 challenge (Supplemental File 2), where in some mice an increase in frequency of these cells over time was observed, likely because of an increase in reconstitution in the absence of HIV-1 infection. Thus, in a direct comparison between Hu-mice under the influence of the synthetic progestin DMPA versus Hu-mice in the diestrus/progesterone high phase of the estrous cycle, DMPA was found to significantly enhance HIV-1 infection in humanized mice following IVAG exposure (3.25 aOR). This suggests that DMPA increases the risk of HIV-1 acquisition in Hu-mice even more than the endogenous progesterone in the estrous cycle does.

**Table 1:** The association between HIV-1 infection in humanized mice following intravaginal viral challenge and the listed covariables was estimated in a univariate (unadjusted) and multivariate (adjusted) generalized linear model. *P* ≤ 0.05 was considered significant.

**Generalized linear model of infection status gives the probablility that a humanized mouse (N = 95) is infected with HIV-1 following intra-vaginal challenge, based on the variables listed**				
**Variable**	**Unadjusted OR (95% CI)**	***P***	**Adjusted OR (95% CI)**^**a**^	***P***
DMPA (yes)	2.50 (1.09–5.88)	***0.032***	3.25 (1.28–8.86)	***0.016***
CD45 (> 10%)	2.92 (1.25–7.11)	***0.015***	3.40 (1.30–9.58)	***0.015***
Viral dose (high)	2.94 (1.29–6.89)	***0.011***	2.21 (0.91–5.45)	0.082

Bold and italicized values indicates statistically significant odds ratios (ORs)

^a^Adjusted by DMPA, CD45, and Dose.

### DMPA extends the window of susceptibility to HIV-1 infection in humanized mice

**Figure 3:**
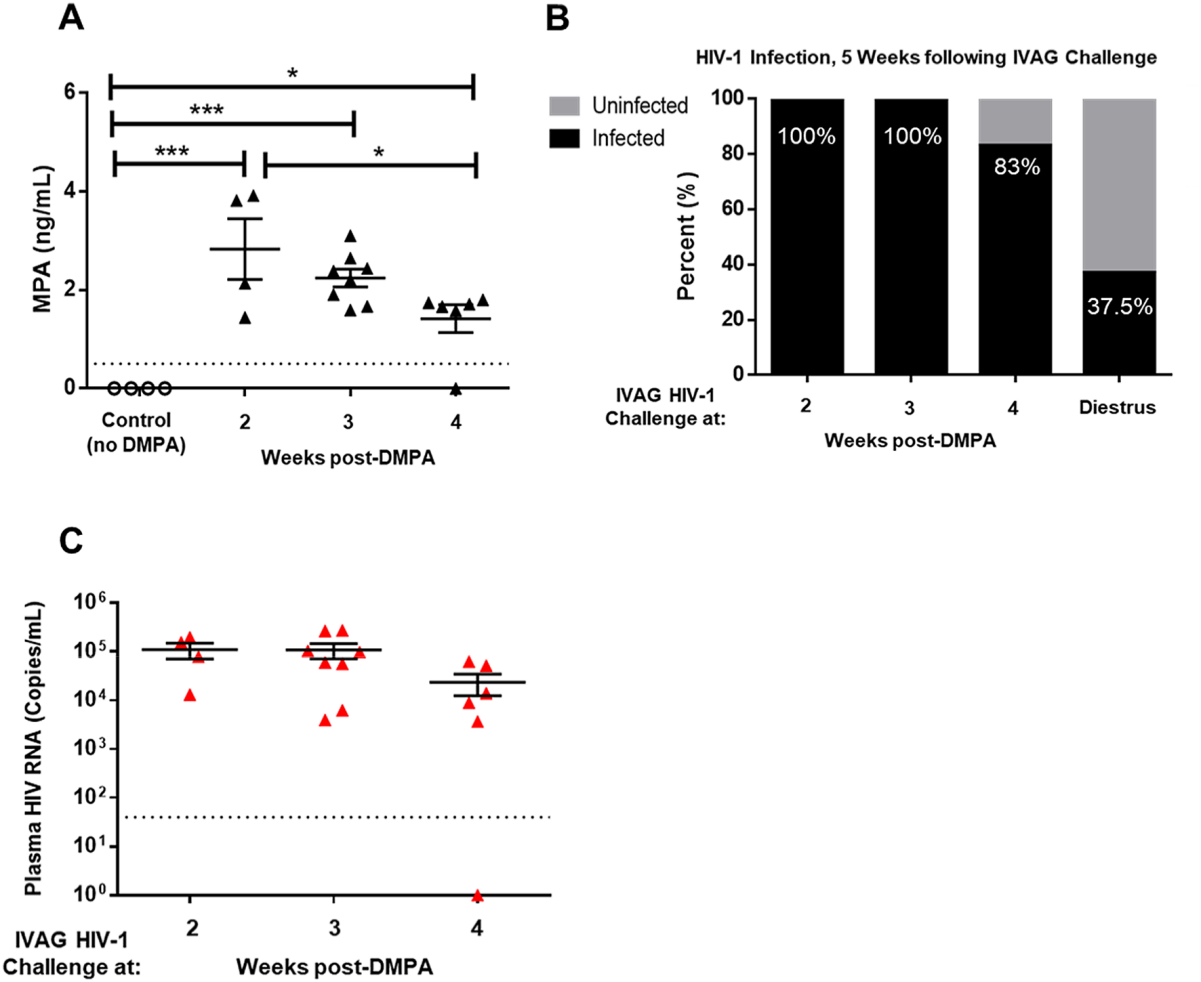
DMPA extends the window of susceptibility to HIV-1 infection in humanized mice. Hu-mice were treated with 2 mg subcutaneous DMPA and received IVAG challenge with HIV-1 at 2, 3, or 4 weeks post-DMPA (N = 18) to determine if the window of HIV-1 susceptibility was extended in DMPA-treated Hu-mice than cycling Hu-mice which were only susceptible to HIV-1 during diestrus (~ 50% of the estrous cycle). (**A**) To confirm that Hu-mice were under the influence of DMPA, MPA, the active ingredient in DMPA, was quantified in the serum. MPA decreased between 2 and 4 weeks post-DMPA, and the amount of MPA was significantly greater in DMPA-treated Hu-mice than controls (N = 4) that had not received DMPA (2.8 ± 0.6 at 2 weeks, 1.4 ± 0.3 at 4 weeks, and 0.0 ± 0.0 ng/mL in controls; *P* < 0.0001, one-way ANOVA). (**B**) Hu-mice were challenged IVAG with HIV-1 at 2, 3, and 4 weeks post-DMPA according to Supplemental Fig. 1. At 2 (N = 4) and 3 (N = 8) weeks post-DMPA 100% of the Hu-mice were susceptible to HIV-1 infection, while at 4 weeks post-DMPA (N = 6) 83% of Hu-mice were susceptible. In comparison, only 37.5% of Hu-mice challenged during diestrus were susceptible to HIV-1 infection (Fig. 2). (**C**) Plasma viral titres were compared between groups, at 5 weeks post-challenge, and the titres were not significantly different. Hu-mice challenged 2, 3, or 4 weeks post-DMPA had equivalent HIV-1 titres at 5 weeks post-infection (1.1 × 10^5^ ± 4.0 × 10^4^, 1.1 × 10^5^ ± 3.8 × 10^4^, 2.8 × 10^4^ ± 1.2 × 10^4^ HIV-1 RNA copies/mL respectively; *P* = 0.1497 one-way ANOVA). *P ≤ 0.05. ***P ≤ 0.001. Data in A and C is presented as mean ± SEM, data in B is presented as proportions (percent infected). *DMPA* Depot-medroxyprogesterone acetate, *IVAG* intravaginal, *MPA* medroxyprogesterone acetate (active ingredient of DMPA).

Previous studies have shown that DMPA increased viral susceptibility by prolonging the period of vulnerability to HSV-2 in vivo^24^. Therefore, we next sought to determine if one of the mechanisms by which DMPA increased susceptibility to HIV-1 was by extending the period of susceptibility to infection. As above, Hu-mice were reconstituted with hCD34 hematopoietic stem cells as described in Methods and “humanization” (%hCD45+ cells in peripheral blood) was assessed 90–120 days later by flow cytometry (Supplemental Fig. 2; Supplemental File 1). We administered 2 mg subcutaneous DMPA and exposed Hu-mice IVAG to HIV-1 (NLR4.3-Bal-Env) at 2 (N = 4), 3 (N = 8), or 4 (N = 6) weeks post-administration of DMPA (Supplemental Fig. 1) to determine the length of time the mice remained susceptible to HIV-1. To ensure that the experiments were physiologically relevant with respect to hormone levels in serum, we quantified serum MPA, the active ingredient in DMPA, in the Hu-mice at the time of IVAG challenge (2, 3, and 4 weeks post-DMPA administration) (Fig. 3A), as in our prior study^14^. In all groups of Hu-mice, the levels of serum MPA were found to be similar to levels observed during the plateau phase of circulating MPA concentrations observed in women^13^. The serum levels of MPA decreased significantly between 2 and 4 weeks post-DMPA in treated mice, while control mice (not given DMPA) had no detectable MPA. When Hu-mice were inoculated IVAG with HIV-1 (10^5^) at 2 (N = 4) and 3 (N = 8) weeks post-DMPA, 100% of the Hu-mice were susceptible to infection (Fig. 3B), while at 4 (N = 6) weeks post-DMPA 83% of Hu-mice were susceptible. From the previous experiment, 37.5% of Hu-mice challenged during diestrus were susceptible to HIV-1 infection (Fig. 2A). This suggests that DMPA-treated Hu-mice are susceptible to IVAG HIV-1 infection for a prolonged period of time compared to control Hu-mice, which are only susceptible to infection during diestrus (Fig. 2A), which lasts 2–3 days in the reproductive cycle of mice. Taken together, this suggests Hu-mice are continuously susceptible to HIV-1 for more than 4 weeks following the administration of DMPA, compared to mice in diestrus that are only susceptible for 50% of the estrous cycle.

At 5 weeks post-challenge plasma viral titres were comparable between groups of Hu-mice treated with DMPA 2 (N = 4), 3 (N = 8), or 4 (N = 6) weeks prior to HIV-1 challenge and no significant differences were observed (1.1 × 10^5^ ± 4.0 × 10^4^, 1.1 × 10^5^ ± 3.8 × 10^4^, 2.8 × 10^4^ ± 1.2 × 10^4^ HIV-1 RNA copies/mL respectively; *P* = 0.1497 one-way ANOVA) (Fig. 3C). Thus, while DMPA extended the window of susceptibility, it did not affect HIV-1 serum titres 5 weeks post-infection.

### DMPA lowers HIV-1 titres in vaginal lavage, and increases activated target cells in peripheral blood of humanized mice

**Figure 4:**
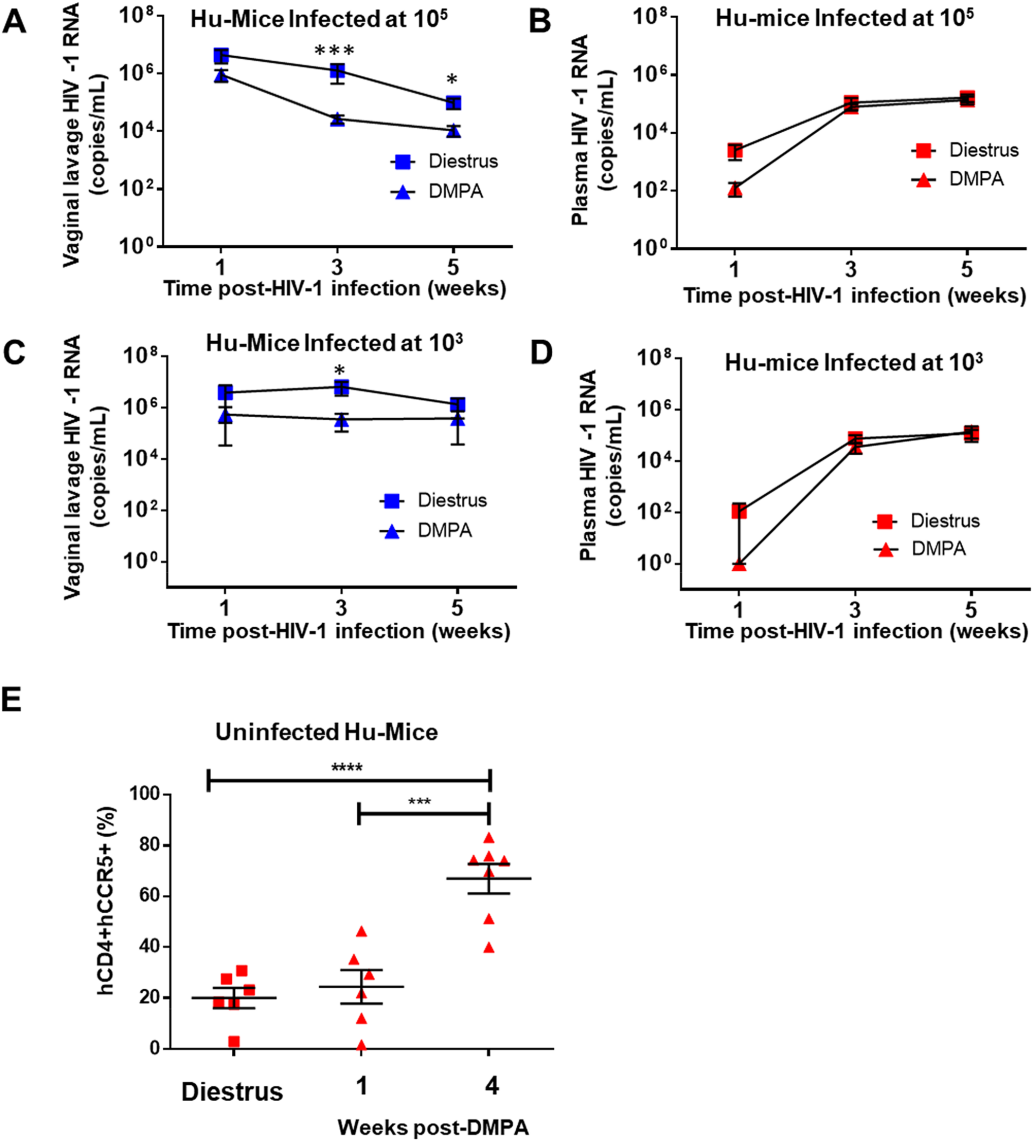
DMPA lowers HIV-1 titres in vaginal lavage, and increases activated target cells in peripheral blood. (**A**) Viral kinetics were tracked by clinical real-time PCR over time in the vaginal lavage of Hu-mice infected with 10^5^ (high dose) HIV-1 during diestrus (N = 14) or following 2 mg DMPA (N = 15). DMPA-treated Hu-mice infected with HIV-1 had significantly lower viral titres in the vaginal lavage at 3 (2.7 × 10^4^ ± 8.5 × 10^3^ DMPA vs. 1.3 × 10^6^ ± 8.3 × 10^5^ copies/mL Diestrus; *P* = 0.0005 Mann–Whitney) and 5 weeks (1.1 × 10^4^ ± 4.3 × 10^3^ DMPA vs. 9.8 × 10^4^ ± 3.9 × 10^4^ copies/mL Diestrus; *P* = 0.0420 Mann–Whitney) post-IVAG infection than Hu-mice infected during diestrus. (**B**) No significant difference in plasma titres was observed 1, 3, or 5 weeks following 10^5^ IVAG HIV-1 infection in Hu-mice at diestrus (N = 14) or following 2 mg DMPA (N = 15). (**C**) Viral kinetics were tracked by clinical real-time PCR over time in the vaginal lavage of Hu-mice infected with 10^3^ (low dose) HIV-1 during diestrus (N = 8) or following 2 mg DMPA (N = 9). DMPA-treated Hu-mice infected with 10^3^ HIV-1 had significantly lower viral titres in the vaginal lavage at 3 (3.5 × 10^5^ ± 2.3 × 10^5^ DMPA vs. 6.5 × 10^6^ ± 3.5 × 10^6^ copies/mL Diestrus; *P* = 0.0109 Mann–Whitney) weeks post-IVAG infection than Hu-mice infected during diestrus. (**D**) No significant difference in plasma titres was observed 1, 3, or 5 weeks following 10^3^ IVAG HIV-1 infection in Hu-mice at diestrus (N = 8) or following 2 mg DMPA (N = 9). (**E**) Flow cytometry was performed on the peripheral blood of uninfected Hu-mice to determine the effect of DMPA on activated circulating HIV-1 target cells (hCD45+hCD3+hCD4+hCCR5+). There were significantly more activated target cells in DMPA-treated Hu-mice at 4 weeks (N = 7) as compared to 1 week post-DMPA (N = 6) or diestrus (N = 6) (66.9 ± 5.8% 4 weeks post-DMPA vs. 24.5 ± 6.6% 1 week post-DMPA vs. 20.1 ± 4.0% diestrus; *P* < 0.0001 one-way ANOVA). The 1 week timepoint corresponds to the time we typically challenge Hu-mice with HIV-1, while the 4 week timepoint corresponds to week 3 of infection, if the Hu-mice had been challenged and infected. *P ≤ 0.05. ***P ≤ 0.001. Data are presented as mean ± SEM. *DMPA* Depot-medroxyprogesterone acetate, *Hu-mice* humanized mice.

After determining DMPA-treated Hu-mice had greater odds of becoming infected with HIV-1 than Hu-mice challenged during diestrus, we sought to determine if viral shedding in the vaginal lavage and/or peripheral blood were affected by administration of DMPA. Our experimental designs are presented in Supplemental Fig. 1. Viral kinetics were tracked over time (at 1, 3, and 5 weeks post-HIV-1 infection) in the vaginal lavage and peripheral blood of Hu-mice infected with 10^5^ (high dose) HIV-1 during diestrus (N = 14) and one week following the administration of 2 mg DMPA (N = 15) (Fig. 4A,B). DMPA-treated Hu-mice infected with HIV-1 had significantly lower viral titres in the vaginal lavage at 3 (2.7 × 10^4^ ± 8.5 × 10^3^ DMPA vs. 1.3 × 10^6^ ± 8.3 × 10^5^ copies/mL Diestrus; *P* = 0.0005 Mann–Whitney) and 5 weeks (1.1 × 10^4^ ± 4.3 × 10^3^ DMPA vs. 9.8 × 10^4^ ± 3.9 × 10^4^ copies/mL Diestrus; *P* = 0.0420 Mann–Whitney) post-IVAG infection than Hu-mice infected during diestrus (Fig. 4A). Conversely, no significant difference in plasma titres were observed 1, 3, or 5 weeks following IVAG HIV-1 infection (Fig. 4B). To determine if the HIV-1 suppression seen in Hu-mice infected following DMPA treatment was altered by viral dose at infection, viral kinetics were tracked over time in the vaginal lavage and peripheral blood of Hu-mice infected with 10^3^ (low dose) HIV-1 during diestrus (N = 8) and one week following the administration of 2 mg DMPA (N = 9) (Fig. 4C,D). Similar to what we observed in DMPA-treated Hu-mice infected with 10^5^ HIV-1, DMPA-treated Hu-mice infected with 10^3^ HIV-1 had significantly lower viral titres in the vaginal lavage at 3 weeks post-IVAG infection than Hu-mice infected during diestrus (3.5 × 10^5^ ± 2.3 × 10^5^ DMPA vs. 6.5 × 10^6^ ± 3.5 × 10^6^ copies/mL diestrus; *P* = 0.0109 Mann–Whitney) (Fig. 4C), while plasma titres remained equivalent (Fig. 4D). Taken together, these results suggest that viral titres are suppressed in the vaginal tract during early infection in DMPA-treated Hu-mice, while plasma titres remain unaffected. These data indicate that although Hu-mice under the influence of DMPA are more susceptible to HIV-1, they initially (1–3 weeks post-infection) have lower vaginal viral titres (viral replication) compared to Hu-mice infected during diestrus.

Based on our titre data, we speculated that viral escape from the initial point of infection in the vaginal tract into the systemic circulation and peripheral tissues might be delayed in DMPA-treated Hu-mice, compared to mice infected during diestrus. As plasma titres were not significantly different between groups, we hypothesized that HIV-1 might escape the vaginal tract and disseminate more rapidly in DMPA-treated Hu-mice, perhaps due to increased HIV-1 target cells in blood. Therefore, we performed flow cytometry on the peripheral blood of uninfected Hu-mice to determine the effect of DMPA on activated, circulating HIV-1 target cells (hCD45+hCD3+hCD4+hCCR5+) (Fig. 4E). When target cells in the peripheral blood of uninfected Hu-mice in diestrus (N = 6) were compared to uninfected Hu-mice 1 week (corresponds to the time we typically challenge Hu-mice with HIV-1; N = 6) and 4 weeks (corresponds to week 3 of infection, if the Hu-mice had been infected; N = 7) following DMPA, there were significantly more hCD45+hCD3+hCD4+hCCR5+ target cells in the peripheral blood of DMPA-treated Hu-mice at 4 weeks than 1 week post-DMPA or diestrus (66.9 ± 5.8% 4 weeks post-DMPA vs. 24.5 ± 6.6% 1 week post-DMPA versus 20.1 ± 4.0% diestrus; *P* < 0.0001 one-way ANOVA). This suggests that in the absence of HIV-1 infection, DMPA enhances circulating HIV-1 target cells in the peripheral blood 4 weeks after administration. In the presence of HIV-1 infection, we observed the classical decline in the proportion of hCD45+hCD3+hCD4+hCCR5+ HIV-1 target cells in the vagina and peripheral blood following HIV-1 infection (Supplemental Fig. 3). The proportion of hCD45+hCD3+hCD4+hCCR5+ cells in the vagina and peripheral blood were quantified by flow cytometry, and Hu-mice that had been intravaginally infected with HIV-1 had significantly less frequency of hCD45+hCD3+hCD4+hCCR5+ cells in the vagina and peripheral blood cells than uninfected Hu-mice (vagina: 12.0 ± 1.1% 5 weeks post-intravaginal HIV-1 infection (N = 15) vs. 63.2 ± 5.5% in uninfected Hu-mice (N = 23); *P* < 0.0001 Mann–Whitney test; peripheral blood: 12.0 ± 1.1% 5 weeks post-intravaginal HIV-1 infection (N = 15) vs. 63.2 ± 5.5% in uninfected Hu-mice (N = 22); *P* < 0.0001 Mann–Whitney test).

In order to further support our hypothesis that there is initially a delay in HIV-1 escape from vaginal tract into the systemic circulation in DMPA-treated Hu-mice versus those infected during diestrus we examined the extent of viral dissemination. Clinical real-time PCR for HIV-1 RNA was performed on homogenates (organ or tissue), and body fluids collected from Hu-mice infected during diestrus (N = 4) or following treatment with DMPA (N = 4) at 1 and 5 weeks post-infection, as described in Materials and Methods (Supplemental Fig. 1). Hu-mice infected during diestrus had more extensive viral dissemination 1 week after infection than DMPA-treated Hu-mice (Supplemental Fig. 4A,B). However, by 5 weeks post-infection DMPA-treated Hu-mice had similar viral dissemination as Hu-mice infected during diestrus (Supplemental Fig. 4C,D), possibly due to the increased hCD45+hCD3+hCD4+hCCR5+ target cells we observed in the peripheral blood (Fig. 4E). Taken together, our results suggest that DMPA initially suppresses local HIV-1 titres in the vaginal tract, and delays systemic viral spread. However, in the peripheral blood, DMPA appears to increase activated target cells over time, which may allow for comparable viral levels to those Hu-mice infected during diestrus, by 5 weeks post-infection.

### DMPA does not alter inflammatory cytokines in the vaginal tract of Hu-mice

**Figure 5:**
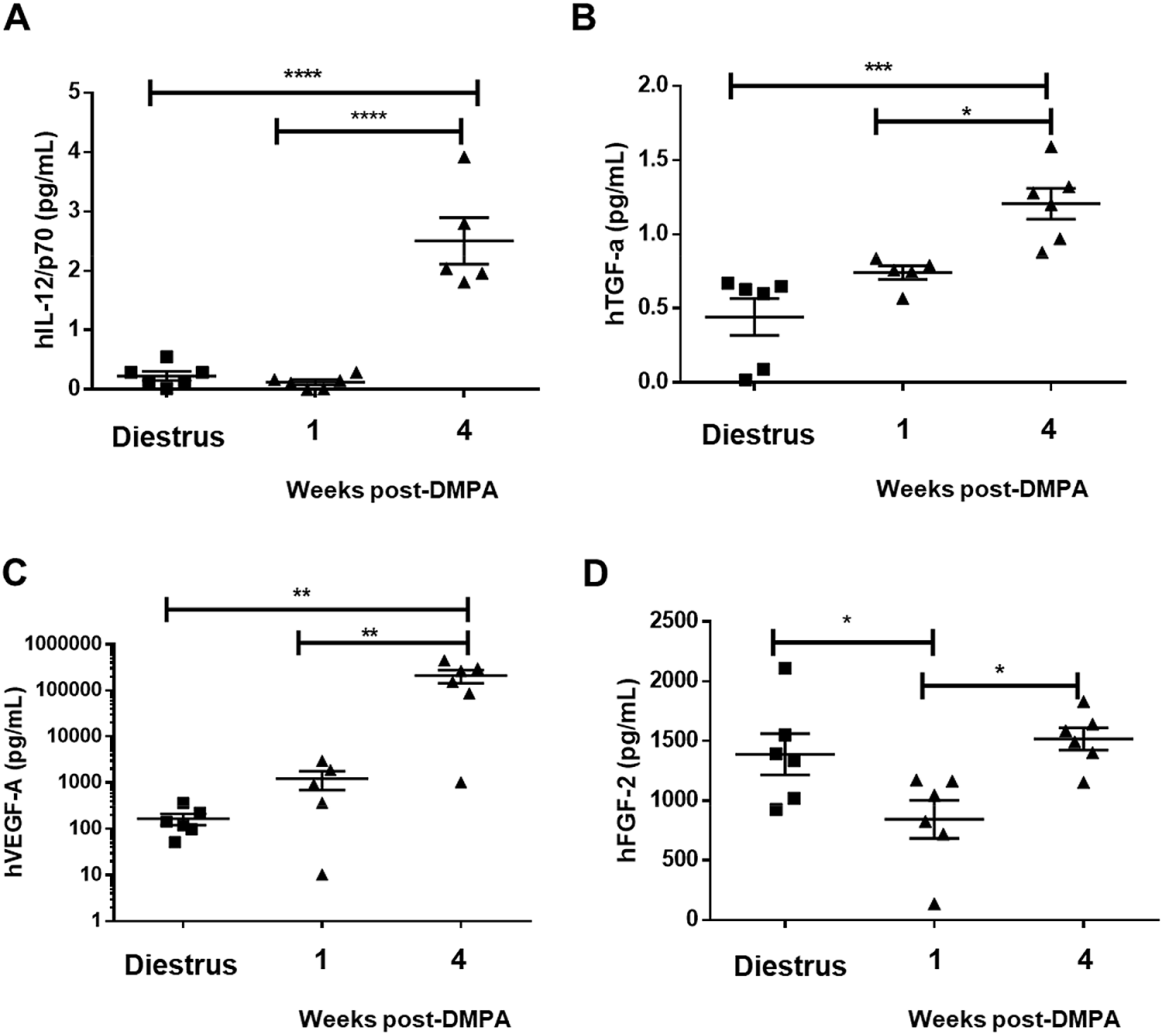
DMPA alters vaginal cytokines in Hu-mice. Cytokines were quantified in the vaginal homogenates of uninfected (never challenged with HIV-1) Hu-mice in diestrus (N = 6) and at 1 week (N = 6), or 4 weeks post-DMPA (N = 6) to determine if DMPA altered vaginal cytokines. The 1 week timepoint corresponds to the time we typically challenge Hu-mice with HIV-1, while the 4 week timepoint corresponds to week 3 of infection, if the Hu-mice had been challenged and infected. (**A**) hIL12/p70 (*P* ≤ 0.0001 one-way ANOVA), (**B**) hTGF-α (*P* = 0.0003 one-way ANOVA), and (**C**) hVEGF-A (*P* = 0.0029 one-way ANOVA) were significantly greater in Hu-mice at 4 weeks post-DMPA as compared to 1 week post-DMPA and diestrus. (**D**) hFGF-2 (*P* = 0.0125 one-way ANOVA) was significantly suppressed at 1 week post-DMPA as compared to 4 weeks post-DMPA and diestrus. *P ≤ 0.05. **P ≤ 0.01. ***P ≤ 0.001. ****P ≤ 0.0001. Data are presented as mean ± SEM. *DMPA* Depot-medroxyprogesterone acetate.

After determining that DMPA increased infection compared to Hu-mice challenged IVAG with HIV-1 during diestrus, and that DMPA suppressed viral titres in the vaginal washes and initially slowed viral dissemination, we wanted to quantify a panel of cytokines and chemokines in the vaginal homogenates of uninfected Hu-mice that had received DMPA or not (diestrus). A total of 23 human cytokines/chemokines were quantified in the vaginal homogenates of uninfected Hu-mice in diestrus (N = 6), 1 week post-DMPA (N = 6), or 4 weeks post-DMPA (N = 6; time point would correspond to 3 weeks post-infection if the Hu-mice had been infected). Of the 23 human cytokines/chemokines quantified, 4 were significantly different between Hu-mice in diestrus as compared to those treated with DMPA (Fig. 5), with the greatest differences occurring between Hu-mice in diestrus versus 4 weeks post-DMPA. Three cytokines, hIL12/p70 (Fig. 5A; *P* ≤ 0.0001 one-way ANOVA), hTGF-α (Fig. 5B; *P* = 0.0003 one-way ANOVA), and hVEGF-A (Fig. 5C; *P* = 0.0029 one-way ANOVA), were significantly greater in Hu-mice at 4 weeks post-DMPA as compared to those at 1 week post-DMPA and those in diestrus, while one cytokine, hFGF-2 (Fig. 5D; *P* = 0.0125 one-way ANOVA), was significantly suppressed at 1 week post-DMPA as compared to 4 weeks post-DMPA and diestrus. No significant differences were observed for the other cytokines/chemokines quantified including hIL-1β (*P* = 0.2427), hIL-4 (*P* = 0.1995), hIL-5 (*P* = 0.9854), hIL-6 (*P* = 0.1713), hIL-8 (*P* = 0.1146), hIL-9 (*P* = 0.9854), hIL-10 (*P* = 0.0655), hIL-13 (*P* = 0.5273), hTNF-α (*P* = 0.2085), hIFN-γ (*P* = 0.0658), hMCP-1 (*P* = 0.3431), hMCP-3 (*P* = 0.3935), hGM-CSF (*P* = 0.2697), hCX3CL (*P* = 0.2720), hCXCL1 (*P* = 0.5866), hCCL22 (*P* = 0.3168), hsCD40L (*P* = 0.0716), and hFlt-3L (*P* = 0.1123) (Supplemental Fig. 5). Human IL-2 was below the assay limit of detection and could not be quantified in the Hu-mouse vaginal homogenates.

We also quantified 10 murine cytokines/chemokines in the vaginal homogenates of uninfected Hu-mice in diestrus (N = 6), 1 week post-DMPA (N = 3), or 4 weeks post-DMPA (N = 3; time point would correspond to 3 weeks post-infection if the Hu-mice had been infected). No significant differences in murine cytokines/chemokines quantified were observed between Hu-mice in diestrus as compared to those treated with DMPA (Supplemental Fig. 6) for mIL-1β (*P* = 0.2479), mIL-2 (*P* = 0.8655), mIL-6 (*P* = 0.4051), mIL-10 (*P* = 0.4987), mIL-12/p70 (*P* = 0.5248), mGM-CSF (*P* = 0.2909), mMCP-1 (*P* = 0.7291), or mTNF-α (*P* = 0.4667). Murine IL-4 and IFN-γ were below the assay limit of detection and could not be quantified in the Hu-mouse vaginal homogenates.

These results suggest that the greatest effect of DMPA on human vaginal cytokines occurs at 4 weeks post-DMPA rather than 1 week post-DMPA, and that the cytokines/chemokines we quantified are likely not contributing to the increased HIV-1 infection we see in the present study, in Hu-mice given DMPA (infected at 1 week post-DMPA) compared to Hu-mice in diestrus.

### Macrophages are increased in the vaginal tract of DMPA-treated Hu-mice

**Figure 6:**
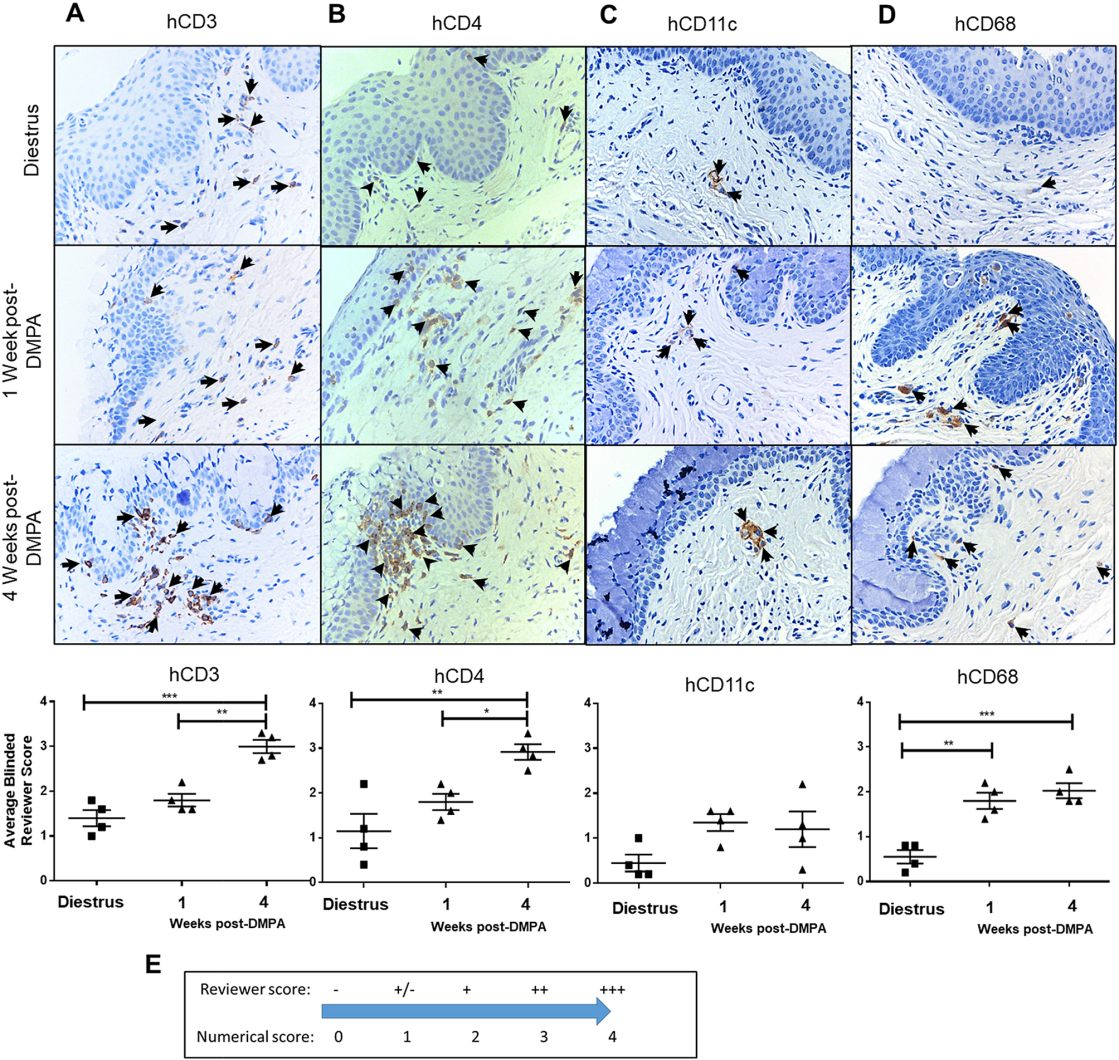
Macrophages are initially enhanced in the vaginal tract of DMPA-treated Hu-mice. Immunohistochemical staining (brown stain, arrowheads) for HIV-1 target cells (**A**) hCD3, (**B**) hCD4, (**C**) hCD11c (DCs), and (**D**) hCD68 (macrophages) was performed on the vaginal mucosa of uninfected Hu-mice in diestrus (N = 5), 1 week (N = 6), and 4 weeks post-DMPA (N = 5). Slides were independently scored by 4 reviewers blinded to treatment, according to the scoring continuum (**E**). DMPA treatment enhanced hCD3 (**A**) (*P* = 0.0001, one-way ANOVA), hCD4 (**B**) (*P* = 0.0036, one-way ANOVA), and (**D**) hCD68 populations (*P* = 0.0003, one-way ANOVA), while hCD11c (**C**) remained constant (*P* = 0.0959, one-way ANOVA). The only HIV-1 target cell population that was significantly different between Hu-mice in diestrus and 1 week post-DMPA (time point corresponding to when we would typically challenge Hu-mice with HIV-1) was hCD68+ cells, suggesting DMPA-treated Hu-mice are initially more susceptible to intravaginal infection with HIV-1 due to increased vaginal macrophages. *P ≤ 0.05. **P ≤ 0.01. ***P ≤ 0.001. Data are presented as mean ± SEM. *DMPA* Depot-medroxyprogesterone acetate.

Although there were no major differences in the cytokines and chemokines quantified between uninfected (never exposed) Hu-mice in diestrus and 1 week post-DMPA (time point when we would typically challenge Hu-mice IVAG with HIV-1), we sought to examine the populations of HIV-1 target cells (T cells, DCs, and macrophages) in the vaginal mucosa of uninfected Hu-mice. Immunohistochemical staining for hCD3 (T cells), hCD4 (T helper cells), hCD11c (DCs), and hCD68 (macrophages) was performed on the vaginal mucosa of uninfected Hu-mice in diestrus (N = 5), 1 week (N = 6), and 4 weeks post-DMPA (N = 5) (Fig. 6). Slides were independently scored by 4 researchers who were blinded to treatment status. Researcher scores were translated into numerical scores according to the scoring continuum (Fig. 6E).

DMPA treatment enhanced hCD3 (Fig. 6A; *P* = 0.0001, one-way ANOVA), hCD4 (Fig. 6B; *P* = 0.0036, one-way ANOVA), and hCD68 populations (Fig. 6D; *P* = 0.0003, one-way ANOVA) by 4 weeks post-DMPA in the uninfected Hu-mouse vaginal tract, while hCD11c remained constant (Fig. 6C; *P* = 0.0959, one-way ANOVA). However, the only HIV-1 target cell population that was significantly different between Hu-mice in diestrus and 1 week post-DMPA (time point corresponding to when we would typically challenge Hu-mice with HIV-1) was hCD68+ cells. This suggests that DMPA-treated Hu-mice may have been more susceptible to intravaginal infection with HIV-1 due to increased macrophages present in the vaginal mucosa at 1 week post-DMPA. The hCD3 and hCD4 populations are enhanced by DMPA, but this only occurs at 4 weeks post-DMPA (time point that would correspond to ‘3 weeks post-infection’ if the Hu-mice had been infected). Thus, enhanced vaginal macrophages might explain why DMPA-treated mice are more susceptible to HIV-1 (1 week post-DMPA) as compared to Hu-mice in diestrus (Fig. 2A, Table 1). Taken together we propose DMPA-treated Hu-mice may initially be more susceptible to HIV-1 as a result of an increase in the vaginal macrophage population.

## Discussion

Herein we demonstrated that DMPA, a synthetic progestin, alters the integrity of the vaginal epithelium, and increases the proportion of Hu-mice infected with HIV-1 compared to Hu-mice challenged during diestrus (progesterone high phase of the estrous cycle). We also show that DMPA treatment increases the window of HIV-1 susceptibility since Hu-mice are continuously susceptible up to 4 weeks post-DMPA, while untreated Hu-mice are only susceptible during diestrus, which represents about 50% of the murine estrous cycle. Interestingly, we found that at that 1 week post-DMPA; the time we would typically challenge Hu-mice with HIV-1, CD68+ macrophages were increased in the vaginal tract of DMPA-treated Hu-mice, even though conventional HIV-1 target cells (CD3+CD4+ T cells) were not increased. Taken together, we posit that the initial increase in HIV-1 susceptibility in DMPA treated Hu-mice could be due to the presence of macrophages which become infected but do not support the robust replication of HIV-1 in the vaginal tract. Subsequently by 3–4 weeks post-DMPA treatment, increased target T cells in the vagina may enhance active viral replication, allowing dissemination and replication of HIV-1 in peripheral blood, such that viral kinetics were similar between Hu-mice infected with HIV-1 following DMPA and those infected during diestrus by 5 weeks post-infection. A tabular summary of our study results can be found in Table 2, and our experimental designs are presented in Supplemental Fig. 1.

**Table 2:** A summary of the study results.

	Diestrus	DMPA
**Vaginal epithelial barrier function**		
Desmoglein-1α staining	↓	↑
FITC-dextran dye leakage	↓	↑
**HIV-1 infection rate and susceptibility**		
HIV-1 infection	↓	↑
Window of HIV-1 susceptibility	↓	↑
**HIV-1 titres**		
Vaginal HIV-1 titres	↑	↓
Blood HIV-1 titres	=	=
**HIV-1 dissemination**		
Viral dissemination (early infection)	↑	↓
Viral dissemination (late infection)	=	=
**HIV-1 target cells**		
Vaginal hCD4+ Cells (1 week post-DMPA)	=	=
Vaginal hCD4+ Cells (4 week post-DMPA)	↓	↑
Vaginal hCD11c+ Cells (1 week post-DMPA)	=	=
Vaginal hCD11c+ Cells (4 week post-DMPA)	=	=
Vaginal hCD68+ Cells (1 week post-DMPA)	↓	↑
Vaginal hCD68+ Cells (4 week post-DMPA)	↓	↑
Blood hCD4+CCR5+ Cells (1 week post-DMPA)	=	=
Blood hCD4+CCR5+ Cells (4 week post-DMPA)	↓	↑

An upward arrow (↑) indicates the factor of interest was significantly greater in that group (Diestrus vs. DMPA), while an equals sign (=) indicates groups were similar, and a downward arrow (↓) indicates the factor of interest was significantly lower.

The female sex hormones estradiol and progesterone, and their synthetic derivatives, found in hormonal contraceptives, are associated with changes in susceptibility to STIs including HIV-1, likely via a variety of biological mechanisms (reviewed in^13^). Typically, estradiol is associated with protection from HIV-1 in women, while progesterone and DMPA have been thought to increase the risk of infection^9,10,25–27^, and similar phenomena are reported in non-human primate (NHP) model systems^5,6,8,28^. One of the mechanisms by which the female sex hormones are thought to modify the risk of HIV-1 infection is via the modification of target cells. Indeed, estradiol has been shown to reduce susceptibility of human peripheral blood CD4+ T cells and macrophages to HIV-1^29^ in vitro. Interestingly, in the same study the protective effect of estradiol against HIV-1 infection was more pronounced in macrophages than in CD4+ T cells. In a humanized mouse model (NOD-scid-IL-2Rgc−/− mice with human peripheral blood mononuclear cells—no tissue reconstitution), different from the Hu-mouse model used in the present study (where we have tissue reconstitution with human immune cells^15^), Quispe Calla et al. ^30^ demonstrated that when control mice were challenged during estrus (when estradiol is high) they were not susceptible to IVAG HIV-1, similar to our findings (Fig. 2A). Their study also demonstrated that Hu-mice treated with DMPA were susceptible to IVAG infection, but could be protected when concurrently treated with estradiol^30^, suggesting that the presence of estradiol is necessary to protect against HIV-1 infection in Hu-mice. Additionally, our previous paper also demonstrated that DMPA enhanced the risk of HIV-1 infection in Hu-mice^14^, and herein we assessed the mechanisms by which this occurs. As DMPA is potently anti-estrogenic; women on DMPA are hypo-estrogenic (reviewed in^13^), it is perhaps this lack of estrogen that increases the susceptibility of vaginal macrophages in our DMPA-treated mice to HIV-1 and results in an increased infection rate as compared to Hu-mice challenged during diestrus (where there would still be some estradiol in the circulation). Furthermore, we found that the integrity of the vaginal epithelial barrier was greatest in mice in estrus (when estradiol is highest), compared with those in diestrus or treated with DMPA (when estradiol is lower) (Fig. 1). This suggests that estradiol likely protects Hu-mice against HIV-1 by multiple mechanisms.

While estradiol is often found to protect against STIs, progesterone, and in particular DMPA, is generally thought to enhance the risk of HIV-1 acquisition. Indeed, meta-analyses have found women on DMPA are 40% more likely to acquire HIV-1 than women not on hormonal contraceptives^11^. Studies in NHPs show that DMPA enhances the risk of vaginal acquisition of SIV and increases viral titres during early infection^21–23^. Similarly, NHPs exposed to HIV-1 during the luteal phase of their menstrual cycle (progesterone high) are more susceptible to vaginal HIV-1 infection than those challenged during the follicular phase, when estrogen is high^5,31^. Here, we were interested in directly comparing and determining if Hu-mice treated with a synthetic progestin (DMPA) were more susceptible to HIV-1 as compared to Hu-mice in diestrus, with naturally elevated progesterone. Diestrus is the stage of the murine reproductive cycle when endogenous progesterone is highest. We found that indeed, Hu-mice challenged 1 week after treatment with 2 mg of DMPA were 3.25 times more susceptible to intravaginal infection with HIV-1 as compared to those challenged during diestrus. This study demonstrates for the first time that DMPA increases susceptibility to HIV-1 in Hu-mice above the naturally occurring window of susceptibility present during the progesterone-high diestrus phase of the estrous cycle (50% of the estrous cycle).

In order to determine the mechanisms by which DMPA enhances susceptibility to HIV-1 we examined the length of the window of vulnerability and prevalence of vaginal HIV-1 target cells. First, Hu-mice treated with DMPA were susceptible to intravaginal infection up to 4 weeks following a single 2 mg dose, while untreated Hu-mice were only susceptible to infection during diestrus. This suggests that one of the mechanisms by which DMPA enhances the risk of acquiring HIV-1 is because it causes an extended state of perpetual viral susceptibility. As compared to Hu-mice in diestrus, which were only susceptible during this phase of the estrous cycle (representing approximately 50% of the length of the mouse estrous cycle), Hu-mice treated with DMPA could be infected following viral exposure at any point in the 4 weeks following treatment. Second, DMPA-treated Hu-mice had a greater abundance of CD68+ macrophages, one of the types of HIV-1 target cells, in the vaginal mucosa as compared to Hu-mice in diestrus, suggesting that DMPA may initially enhance the vaginal macrophage population. This increase in macrophages might explain why DMPA-treated Hu-mice were 3.25× more likely to be infected following intravaginal challenge as compared to Hu-mice in diestrus. In fact, DMPA has previously been reported to enhance macrophage populations in the human vagina^32^ and endometrium^33^. Similarly, DMPA has been demonstrated to significantly increase CD68+ macrophages in the ectocervix and vaginal mucosa of NHPs, even above levels observed during the luteal phase (progesterone high) of the menstrual cycle^8^, suggesting this may be the reason DMPA-treated Hu-mice are more susceptible to HIV-1 infection than those challenged during diestrus (progesterone high).

Although DMPA-treated Hu-mice were more susceptible to HIV-1 infection, unexpectedly they had significantly lower vaginal HIV-1 titres and initial viral dissemination was limited as compared to Hu-mice infected during diestrus, even though plasma HIV-1 titres were comparable. In addition to its anti-estrogenic effect, DMPA is also reported to have immunosuppressive effects, particularly at high doses in humans; however, the threshold below which immunosuppression no longer occurs may vary by individual and/or may also occur at physiologically relevant circulating concentrations of MPA (active ingredient in DMPA) (reviewed in^13^). Unlike progesterone, which only binds progesterone receptors, DMPA binds both progesterone receptors and glucocorticoid receptors (GRs). Although interactions are complex and not easily explained, it is thought that the immunosuppressive effects of DMPA occur due to its ability to bind GRs and repress a multitude of pro-inflammatory pathways (reviewed in^13^). In our previous study^14^ we found that a 2 mg subcutaneous dose of DMPA in Hu-mice (same dose employed in the present study) consistently led to circulating levels of MPA similar to those observed in women; including both a peak and plateau phase. Thus, as DMPA can be immunosuppressive in women at physiologically relevant concentrations, it is likely that these effects would also be seen in Hu-mice, and this might explain why DMPA-treated Hu-mice had significantly lower vaginal HIV-1 titres and initially limited viral dissemination as compared to Hu-mice infected during diestrus. Viral replication in the blood (Fig. 4B,D), and dissemination during late infection (5 weeks post-infection) (Supplemental Fig. 4C,D) was similar between Hu-mice infected with HIV-1 following DMPA and those infected during diestrus, which might be explained by the fact that vaginal T cells (hCD3+, hCD4+) (Fig. 6A,B) and activated circulating HIV-1 target cells (hCD45+hCD3+hCD4+hCCR5+) (Fig. 4E) were greater in DMPA-treated Hu-mice (4 weeks post-DMPA—in the absence of HIV-1 infection) than Hu-mice in diestrus. Enhanced vaginal recruitment of target cells and enhanced target cells in the peripheral blood would allow viral spread in the DMPA-treated Hu-mice to catch up to that observed in diestrus infected Hu-mice. Together our results suggest DMPA initially suppresses local HIV-1 titres in the vaginal tract, and delays viral spread, while enhanced target cells in vaginal tract and the peripheral blood by 5 weeks post-infection allows dissemination to then proceed quickly, rendering it comparable to Hu-mice infected during diestrus.

Other Hu-mouse models have found that administration of DMPA results in loss of genital mucosal barrier function^19^, and enhanced genital transmission of cell-associated HIV (as compared to estradiol treated Hu-mice). NHP models have demonstrated that DMPA-treated macaques have a greater density of HIV-1 target cells (CD4+ and CD68+ cells) in the ectocervix and vagina as compared to untreated animals, and that macaques in the late luteal phase (high endogenous progesterone) also had more vaginal HIV-1 target cells than those in the follicular phase and mid-cycle^8^. Furthermore, increased viral entry into the cervix and decreased vaginal epithelial thickness during progesterone/progestin dominant states were reported in these macaques^8^, and thinned vaginal epithelium and changes in vaginal pH in pigtail macaques treated with DMPA^17^, offering several additional mechanisms by which DMPA may impact susceptibility to SHIV in the NHP model. A thinned vaginal epithelium was also seen in macaques given subcutaneous progesterone implants, and the vaginal transmission of SIV was enhanced 7.7 fold in these animals compared to placebo implants or macaques challenged during the follicular phase of the menstrual cycle^28^. Unlike in our study, Marx et al. found plasma SIV RNA was elevated in progestin-treated macaques for 3 months following infection, and several of the progestin-treated animals developed rapidly spreading disease. Similarly, in a SHIV macaque model, acute viremia was higher in DMPA-treated animals than naïve animals, and the genetic complexity of the replicating virus was greater too^21^. This study also reported dampened cellular immune responses and an immunosuppressive effect of DMPA^21^, similar to what we observed in our Hu-mice. Although we did not see a difference in acute viremia between Hu-mice infected during diestrus versus those infected following DMPA, perhaps this is a reflection of the fact that our control group also had high endogenous progesterone, whilst controls in other studies may not have had been from the progesterone high phase of the cycle, or it could be an inherent difference between Hu-mouse models and NHPs.

In summary, we demonstrate the striking ability of the synthetic progestin DMPA to significantly increase intravaginal HIV-1 infection 3.25× beyond what was observed for endogenous progesterone in Hu-mice. We also found that the window of HIV-1 susceptibility was extended in DMPA-treated Hu-mice. Furthermore, vaginal HIV-1 target cells, in particular vaginal macrophages (hCD68+) are enhanced in DMPA-treated Hu-mice, and may thus play a previously underappreciated but important role in the vaginal acquisition of HIV-1. We know endometrial macrophages are increased in women on DMPA^33^, however the specific effect of DMPA on vaginal macrophages in women is lacking, but warranted based on our study in Hu-mice. We also found that DMPA initially suppresses local HIV-1 titres in the vaginal tract, and delays viral spread, while enhanced activated target cells in the peripheral blood may allow for viral dissemination to catch up by 5 weeks post-infection. Taken together, results suggest DMPA may enhance susceptibility to HIV-1 in Hu-mice by increasing vaginal target cells (including macrophages), and extending the period of time during which Hu-mice were susceptible to infection; mechanisms that might also affect HIV-1 susceptibility in women.

## Methods

### Study approvals

Human cord blood samples used to generate humanized mice were collected with informed and written consent. All experiments involving human cord blood were performed in accordance with the Canadian ethic guidelines and regulations on the use of human tissue for research and approved by the Hamilton Integrated Research Ethics Board (HiREB). All animal experiments were approved by the McMaster University Animal Research Ethics Board (AREB) as per AUP# 14-09-40, and were conducted in accordance with the Canadian Council of Animal Care (CCAC) guidelines. This study was carried out in compliance with the ARRIVE guidelines.

### Immunofluorescent staining

Experimental designs are presented in Supplemental Fig. 1. Female C57BL/6 mice (Charles River Laboratories, Constant, QC, Canada) were estrous cycle staged by pipetting 30µL of PBS into and out of the vaginal tract 5–6 times, and examining vaginal cytology by light microscopy as described previously^34–37^. Mice were sacrificed during estrus (N = 4), diestrus (N = 4), or 1 week following a 2 mg subcutaneous injection of DMPA (N = 4) (Pfizer, Mississauga, Canada), as in Nguyen et al.. Vaginal tissues were embedded and flash frozen in OCT (Fisher Scientific, Ottawa, ON, Canada) and 5 µm tissue sections were mounted on glass slides and fixed in methanol. After 3 PBS washes, sections were incubated 1 h with 10% normal donkey serum (Abcam, Cambridge MA) at 4 °C, washed, incubated overnight at ambient temperature with rabbit anti-desmoglein-1 (clone EPR6766(B)) or monoclonal rabbit IgG isotype control (clone EPR25A, data not shown), washed and incubated with 1 h with AlexaFluor 488-labeled donkey anti-rabbit IgG (all antibodies Abcam). All antibodies were diluted in PBS with 1% BSA and 0.05% Tween 20. Sections were counterstained with DAPI and visualized using an Olympus IX81 inverted microscope (Olympus, Richmond Hill, ON, Canada).

### Functional assessment of in vivo vaginal barrier permeability

Experimental designs are presented in Supplemental Fig. 1. Vaginal permeability was evaluated in C57BL/6 mice by quantifying the amount of fluorescein isothiocyanate dextran (FITC-dextran) that had crossed the vaginal epithelial barrier into the blood. Mice in estrus (N = 4), diestrus (N = 4), and 1 week following a subcutaneous dose of 2 mg of DMPA (N = 4) were anesthetized by ketamine and 625 µg of FITC-dextran (Sigma Aldrich, Oakville, ON, Canada) in saline was pipetted into the vaginal tract. Four hours later, serum was collected by cardiac puncture at sacrifice. Fluorescence intensity was measured using a SpectraMax i3 plate reader (Molecular Devices, Sunnyvale, CA, USA) (excitation, 492 nm; emission, 525 nm) and neat serum levels of FITC were determined from an in-plate standard curve of known FITC-dextran concentrations. Percent FITC-dextran leakage was calculated as the ratio of FITC-dextran in the serum divided by the FITC-dextran delivered intravaginally, as a measure of vaginal barrier permeability.

### Humanized mice

Experimental designs are presented in Supplemental Fig. 1. Humanized mice (Hu-mice) were generated as previously described^15^. Briefly, umbilical cord blood from healthy newborns born at McMaster University Medical Centre was obtained following parental consent, processed by RosetteSep Human Cord Blood Progenitor Enrichment Kit (Stem Cell Technologies, Vancouver, BC, Canada), and cryopreserved until required. Four day old *NOD-Rag1*^*tm1Mom*^* Il2rg*^*tm1Wjl*^ (NRG) mice were sub-lethally irradiated (γ-ray, 300 cGy) and given an intrahepatic injection with 1 × 10^6^ to 2 × 10^6^ CD34-enriched hematopoietic stem cells. The percent human immune cell reconstitution was evaluated by flow cytometry 90–120 days following injection by quantifying expression of hCD45, hCD3, hCD4, as well as mouse CD45 in peripheral blood mononuclear cells (PBMCs), as in Nguyen et al., 2017. These Hu-mice have engraftment of human cells in peripheral blood, and body tissues^15^. The average human immune cell reconstitution of the Hu-mice used in the experiments described herein was 12% hCD45+ cells in the peripheral blood (as shown in the first panel of gating strategy in Supplemental Fig. 1, with % hCD45 shown in the first gate). The Hu-mice used in the experiments herein were between 3 and 6 months of age. The reconstitution levels, HIV-1 dose, peripheral blood (PB) titres, vaginal lavage (VL) titres, and infection status for Hu-mice included in Table 1 are found in Supplemental File 1.

### Intravaginal HIV-1 challenge

Experimental designs are presented in Supplemental Fig. 1. Female Hu-mice were administered a 2 mg subcutaneous injection of DMPA (Pfizer, Mississauga, Canada) one week prior to HIV-1 challenge (N = 55—average human immune reconstitution: 11% hCD45+hCD3+hCD4+ cells in the peripheral blood prior to experiments). Other female Hu-mice were estrous cycle staged as per Nguyen et al., 2017, and challenged with HIV-1 during the diestrus phase (progesterone high) (N = 40—average human immune reconstitution: 12% hCD45+hCD3+hCD4+ cells in the peripheral blood prior to experiments) or estrus phase (estrogen high) (N = 4). Hu-mice were given an IP injection of anesthetic (150 mg ketamine per kg/10 mg xylazine per mL), placed in a supine position, and the vaginal tract was gently swabbed with a modified cotton swab to gently remove vaginal mucus (performed in all mice). The vaginal swabs were checked for blood contamination as an indication of vaginal trauma. No signs of vaginal trauma were noted for any of the Hu-mice in these experiments. Hu-mice were intravaginally inoculated with 25 µL of 10^3^ (low) to 10^5^ (high) TCID_50_/mL of NL4.3-Bal-Env HIV-1, using a pipette. Hu-mice remained supine for approximately 1 h following inoculation. Generation of NL4.3-Bal-Env HIV-1 is described in Nguyen et al., 2017. Another group of Hu-mice (N = 18) were challenged 2, 3, or 4 weeks following DMPA administration to examine the effect of DMPA on the window of HIV-1 susceptibility.

### Quantification of serum MPA

Hu-mice (N = 18) were bled by cheek bleed at 2, 3, or 4 weeks post-DMPA to quantify MPA in duplicate in the serum by ELISA (EuroProxima, Arnhem, Netherlands), as per the manufacturer’s protocol, following modifications (Smith 2014)^38^. Serum was collected from NRG mice (N = 4) that had never been exposed to DMPA as a negative control. The assay limit of detection is 0.5 ng/mL.

### Quantification of HIV-1 titres

Peripheral blood was collected in plasma separator tubes (BD Biosciences, San Jose, CA, USA) by cheek or orbital bleeds at 1, 3, and/or 5 weeks post-HIV-1 challenge. Whole blood was centrifuged at 15 000 RPM for 8 min to isolate plasma. Plasma was subsequently frozen at − 80 °C until required. Vaginal lavage was collected at 1, 3, and/or 5 weeks post-HIV-1 challenge by pipetting 2 × 30 μL of sterile PBS in and out of the vaginal tract 3–4 times. Vaginal lavage was frozen at − 80 °C until required. Plasma and vaginal washes were sent on dry ice to the Mt. Sinai Microbiology Laboratory (Toronto, ON, Canada) for clinical real-time PCR quantification of HIV-1 RNA using the Abbott RealTime HIV-1 m2000 (Abbott Laboratories, Illinois, USA). The sensitivity and specificity of this assay are 91.8% and 100% respectively^39^, and its limit of detection is 40 copies/mL (for more information please see: https://www.molecular.abbott/us/en/products/infectious-disease/realtime-hiv-1-viral-load).

Tissues (heart, rectum, colon, small intestine, kidney, liver, spleen, lung, brain, skeletal muscle, bone marrow, bladder, thymus, mesenteric lymph node, vaginal tract, and uterus) were collected at 1 or 5 weeks following IVAG HIV-1 infection, and homogenized in PBS, using the Gold BulletBlender (Next Advance, Averill Park, NY). Homogenates were centrifuged (8000 RPM, 5 min), and supernatants were stored at − 80 °C until required. Tissue supernatants were sent to the Mt. Sinai Microbiology Laboratory (Toronto, ON, Canada) for clinical real-time PCR quantification of HIV-1 RNA (Supplemental Fig. 4).

### Flow cytometry

Blood was collected by cheek bleed (as above) or at experimental endpoint by cardiac puncture as previously described^40^. PBMCs were recovered from blood following ACK lysis buffer (2 mL, 4 min; then 500 μL 1 min), and neutralized with PBS. The resulting cell preparations were counted on a hemocytometer, resuspended to a final concentration of 1–3 × 10^6^ cells/mL, and stained for flow cytometry.

PBMCs were stained for 30 min with antibodies [hCD45—V450, hCD4—PerCP-Cy5.5, hCD8—PE-Cy7 (BD Biosciences, San Jose, CA, USA); hCD3—BV605, hCCR5—AF647 (Biolegend, San Diego, CA, USA); mCD45—AF700 (eBioscience, San Diego, CA, USA)], following 20 min of Fc receptor blocking (eBioscience, San Diego, CA, USA) on ice. Data were immediately acquired on a BD LSRII flow cytometer (BD Biosciences, Canada) and results were analyzed using FlowJo software (Version 10.0.8) (Treestar, Ashland, OR, USA). Gating strategy is presented in Supplemental Fig. 2.

### Quantification of vaginal cytokines

At endpoint, 1/4 of the vaginal tract (split lengthwise) was collected from uninfected Hu-mice in diestrus (N = 6), 1 week post-DMPA (N = 6), and 4 weeks post-DMPA (N = 6) and mechanically homogenized in PBS as above. Vaginal homogenates were centrifuged and a total of 23 unique human cytokines/chemokines were quantified in duplicate in the supernatants using the Human Cytokine Array Focused 13-plex (HDF13) and Featured Human Cytokine/Chemokine Array 11-plex (HCYTP-11–34) (Eve Technologies, Calgary, AB, Canada).

Murine cytokines/chemokines were quantified in duplicate in the vaginal supernatants from uninfected Hu-mice in diestrus (N = 6), 1 week post-DMPA (N = 3), and 4 weeks post-DMPA (N = 3) using the Mouse Focused 10-plex (MDF10) Discovery Assay (Eve Technologies).

### Immunohistochemistry

The vaginal tracts from uninfected Hu-mice (N = 5 diestrus, N = 6 at 1 week post-DMPA, N = 5 at 4 weeks post-DMPA) were fixed in 10% formalin, processed, and embedded in paraffin. Sections were cut at 4 µm, deparaffinized, and stained following blocking with 5% H_2_O_2_, citrate buffer antigen retrieval, and blocking with 5% normal goat serum in TBS-T (20 min) or the Bond Epitope Retrieval 2 system (Leica Biosystems, Concord, ON, Canada) (20 min). Sections were incubated with (1) rabbit monoclonal anti-human CD3 (ab16669, Abcam, Cambridge, MA, USA), diluted 1:150 in PowerVision IHC Superblocking (Leica Biosystems); (2) mouse monoclonal anti-human CD4 (Leica Biosystems), diluted 1:50 in PowerVision (Leica Biosystems); (3) rabbit monoclonal anti-human CD11c (ab52632, Abcam), diluted 1:500 using 0.1% BSA in PBS as a diluent, and (4) mouse monoclonal anti-human CD68 (KP1, Dako Canada Inc., Burlington, ON), undiluted. The Bond Polymer Refine kit (Leica Biosystems) was employed for CD3 (post-primary step excluded) and CD4 staining on the Bond RX automated slide stainer (Leica Biosystems), while rabbit and mouse Vectastain ABC kits (Vector Labs, Burlington, ON, Canada) were employed for CD11c and CD68. All slides were stained with DAB as a chromogen, and counterstained with hematoxylin. Images were visualized using an Olympus IX81 inverted microscope (Olympus, Richmond Hill, ON, Canada), and captured using the Infinity camera (Lumenera Corp., Ottawa, ON, Canada).

### Statistical analyses

To assess the relationship between DMPA and HIV-1 susceptibility in humanized mice (Table 1) we performed univariate (unadjusted) and multivariate (adjusted) generalized linear models, and a multivariate linear mixed effects model with repeated measures using the glm and lmer functions in the stats and lme4 package in R version 3.2.3^41^. In the univariate model, the odds ratio (OR) and 95% confidence interval (CI) were calculated for each covariate (CD45 > 10%, and viral dose (high)). In the multivariate model, the adjusted odds ratios (aOR) and 95% CIs were estimated after adjusting for covariates. In the multivariate model the adjusted regression coefficients (β) and 95% CIs were calculated. For all statistical models, a *P* < 0.05 was considered statistically significant.

Data were analyzed and graphed using GraphPad Prism 6 (GraphPad Software, La Jolla, CA). Data were considered statistically significant if the *P* values obtained with a t-test, analysis variance of the mean (ANOVA), or the equivalent non-parametric tests if the data was not normally distributed, were < 0.05. Significant differences are noted as *P < 0.05, **P < 0.01, ***P < 0.001, or ****P < 0.0001.

## Supplementary Information

Supplementary Information 1.

Supplementary File 1.

Supplementary File 2.
