# Supplementary material for: Depot medroxyprogesterone acetate (DMPA) enhances susceptibility and increases the window of vulnerability to HIV-1 in humanized mice
Source: Sci Rep. 2021 Feb 16;11:3894. doi: 10.1038/s41598-021-83242-9 (PMC7887257; doi:10.1038/s41598-021-83242-9)

**Title:** Depot Medroxyprogesterone Acetate (DMPA) Enhances Susceptibility and Increases the Window of Vulnerability to HIV-1 in Humanized Mice

**Authors**: Jocelyn M. Wessels^1,2^, Philip V. Nguyen^1,2^, Danielle Vitali^1,2^, Kristen Mueller^1,2^, Fatemeh Vahedi^1,2^, Allison M. Felker^1,2^, Haley A. Dupont^1,2^, Puja Bagri^1,2^, Chris P. Verschoor^2^, Alexandre Deshiere^3^, Tony Mazzulli^4,5,6^, Michel J. Tremblay^3^, Ali A Ashkar^1,2^, and Charu Kaushic^1,2*^

**Affiliations:** ^1^McMaster Immunology Research Centre, Michael G. DeGroote Centre for Learning and Discovery, McMaster University, 1280 Main Street West, Hamilton, Ontario, L8S 4K1, Canada; ^2^Department of Pathology and Molecular Medicine, McMaster University, 1280 Main Street West, Hamilton, Ontario, L8S 4K1, Canada; ^3^Axe des Maladies Infectieuses et Immunitaires, Centre de Recherche du CHU de Québec-Université Laval, Pavillon CHUL, Québec City, Québec, G1V 4G2, Canada; ^4^Public Health Laboratories, Public Health Ontario, Toronto, Ontario, M5G 1V2, Canada; ^5^Mount Sinai Hospital/University Health Network, Department of Microbiology, Toronto, Ontario, M5G 1X5, Canada; ^6^Department of Laboratory Medicine and Pathobiology, University of Toronto, Toronto, Ontario M5S 1A8, Canada.

***Corresponding Author:** Dr. Charu Kaushic, McMaster Immunology Research Centre, Department of Pathology and Molecular Medicine, MDCL Room 4014, McMaster University, 1280 Main Street West, Hamilton, Ontario, Canada, L8S 4K1. Fax (905) 522-6750. Phone (905) 525-9140 x22988. Email: [kaushic@mcmaster.ca](mailto:kaushic@mcmaster.ca)

# Supplemental Figure Legends

**Supplemental Figure 1: Experimental design.** C57BL/6 mice were used to assess integrity of the murine vaginal epithelial barrier by desmoglein-1 immunofluorescence (A) and FITC-dextran leakage assay (B). Humanized mice (C) were used for all other experiments. Briefly, NRG mice were irradiated at 4 days of age, and given an intrahepatic injection of CD34 enriched hematopoietic stem cells isolated from human cord blood. The %hCD45 in the peripheral blood (amount of human reconstitution) was quantified in all mice 90-120 days later by flow cytometry. Then Hu-mice were estrous cycle staged or administered 2mg subcutaneous DMPA. Some Hu-mice were intravaginally (IVAG) inoculated with 10^3^ (low) or 10^5^ (high) TCID_50_/mL of NL4.3-Bal-Env HIV-1. Peripheral blood (PB), vaginal lavage (VL) and body tissues (BT; see Supplemental Figure 2) were collected at timepoints noted. Other Hu-mice were never exposed to HIV-1 (uninfected; never challenged). PB and vaginal tracts were collected from these mice for quantification of target cells and vaginal cytokines (human and mouse).BT: body tissues. DMPA: Depot-medroxyprogesterone acetate. HSC: hematopoietic stem cells. Hu-mice: humanized mice. IHC: immunohistochemistry. IVAG: intravaginal. MPA: medroxyprogesterone acetate. PB: peripheral blood. VL: vaginal lavage.

**Supplemental Figure 2: Flow cytometry gating strategy.** The percent human immune cell reconstitution was assessed by flow cytometry in peripheral blood of all Hu-mice 90-120 days following intrahepatic injection with CD34-enriched hematopoietic stem cells isolated from human cord blood, using the first gate in the left panel. We also performed flow cytometry on the peripheral blood of uninfected Hu-mice to determine the effect of DMPA on activated circulating HIV-1 target cells (hCD45+hCD3+hCD4+hCCR5+) by gating on mCD45/hCD45, hCD3, hCD8/hCD4, and CCR5 as presented here, after gating on lymphocytes and single cells.

**Supplemental Figure 3: Decrease in frequency of hCD4+hCCR5+ cells following HIV-1 infection.** Flow cytometry was performed on the vagina (A) and peripheral blood (B) of uninfected Hu-mice and Hu-mice 5 weeks post-infection. (A) There were significantly less activated target cells (hCD45+hCD3+hCD4+hCCR5) in the vagina of HIV-1 infected Hu-mice 5 weeks following infection (N=15) as compared to uninfected Hu-mice (N=23) (63.2±5.5% in uninfected Hu-mice vs. 12.0±1.1% 5 weeks post-intravaginal HIV-1 infection; P<0.0001 Mann-Whitney test). (B) Similarly, there were significantly less activated target cells (hCD45+hCD3+hCD4+hCCR5) in the peripheral blood of HIV-1 infected Hu-mice 5 weeks following infection (N=15) as compared to uninfected Hu-mice (N=22) (21.6±2.4% in uninfected Hu-mice vs. 14.8±2.1% 5 weeks post-intravaginal HIV-1 infection; P=0.04 Mann-Whitney test). *: P≤0.05. ****: P≤0.0001. Data are presented as mean ± SEM.

**Supplemental Figure 4: DMPA initially limits viral dissemination.** Clinical real-time PCR for HIV-1 RNA was performed on homogenates (organ or tissue), and body fluids collected from Hu-mice infected during diestrus (N=4) or following treatment with DMPA (N=4) at 1 and 5 weeks post-infection. Hu-mice infected during diestrus **(A)** had more extensive viral dissemination 1 week after infection than DMPA-treated Hu-mice **(B)**. By 5 weeks post-infection Hu-mice infected during diestrus **(C)** had similar viral dissemination to DMPA-treated Hu-mice **(D)**. Results suggest DMPA initially suppresses local HIV-1 titres in the vaginal tract, and delays viral spread, while enhanced activated target cells in the peripheral blood allows dissemination to proceed quickly and be comparable to Hu-mice infected during diestrus, by 5 weeks post-infection. DMPA: Depot-medroxyprogesterone acetate. LN: lymph node

**Supplemental Figure 5: Quantification of human cytokines and chemokines in humanized mouse vagina.** Cytokines were quantified in vaginal homogenates of uninfected (never challenged with HIV-1) Hu-mice in diestrus (N=6) and at 1 week (N=6), or 4 weeks post-DMPA (N=6) to determine if DMPA altered human vaginal cytokines. The 1 week timepoint corresponds to the time we typically challenge Hu-mice with HIV-1, while the 4 week timepoint corresponds to week 3 of infection, if the Hu-mice had been challenged and infected. No significant differences were observed for **(A)** hIL-1β (P=0.2427), **(B)** hIL-4 (P=0.1995), **(C)** hIL-5 (P=0.9854), **(D)** hIL-6 (P=0.1713), **(E)** hIL-8 (P=0.1146), **(F)** hIL-9 (P=0.9854), **(G)** hIL-10 (P=0.0655), **(H)** hIL-13 (P=0.5273), **(I)** hTNF-α (P=0.2085), **(J)** hIFN-γ (P=0.0658), **(K)** hMCP-1 (P=0.3431), **(L)** hMCP-3 (P=0.3935), **(M)** hGM-CSF (P=0.2697), **(N)** hCX3CL (P=0.2720), **(O)** hCXCL1 (P=0.5866), **(P)** hCCL22 (P=0.3168), **(Q)** hsCD40L (P=0.0716), and **(R)** hFlt-3L (P=0.1123). Data are presented as mean ± SEM. DMPA: Depot-medroxyprogesterone acetate.

**Supplemental Figure 6: Quantification of murine cytokines and chemokines in humanized mouse vagina.** Cytokines were quantified in vaginal homogenates of uninfected (never challenged with HIV-1) Hu-mice in diestrus (N=6) and at 1 week (N=3), or 4 weeks post-DMPA (N=3) to determine if DMPA altered murine vaginal cytokines. The 1 week timepoint corresponds to the time we typically challenge Hu-mice with HIV-1, while the 4 week timepoint corresponds to week 3 of infection, if the Hu-mice had been challenged and infected. No significant differences were observed for **(A)** mIL-1β (P=0.2479), **(B)** mIL-2 (P=0.8655), **(C)** mIL-6 (P=0.4051), **(D)** mIL-10 (P=0.4987), **(E)** mIL-12/p70 (P=0.5248), **(F)** mGM-CSF (P=0.2909), **(G)** mMCP-1 (P=0.7291), and **(H)** mTNF-α (P=0.4667). Data are presented as mean ± SEM. DMPA: Depot-medroxyprogesterone acetate.

**Supplemental File 1: Details of Hu-mice used in Table 1.** Excel file that contains the reconstitution levels (%hCD45 in peripheral blood (PB) 90-120 days following intrahepatic injection with CD34-enriched hematopoietic stem cells), DMPA status (Yes = 2mg DMPA, No = Diestrus stage of estrous cycle), HIV-1 dose, peripheral blood (PB) titres, vaginal lavage (VL) titres, and infection status for Hu-mice (N=95) included in our regression analysis (Table 1).

**Supplemental File 2: Loss of hCD45+hCD3+ HIV-1 target cells in the peripheral blood of Hu-mice following intravaginal HIV-1 infection.** Excel file that contains the initial reconstitution levels (%hCD45 and %hCD45+hCD3+ cells in peripheral blood (PB) 90-120 days following intrahepatic injection with CD34-enriched hematopoietic stem cells), DMPA status (Yes = 2mg DMPA, No = Diestrus stage of estrous cycle), HIV-1 dose, peripheral blood (PB) titres, vaginal lavage (VL) titres, infection status, and experimental endpoint frequencies of %hCD45 and %hCD45+hCD3+ cells in PB, of Hu-mice that did (rows 2-22) and did not (rows 23-34) become infected following intravaginal HIV-1 challenge. Infected Hu-mice (rows 2-22) demonstrate the classical loss of HIV-1 target cells (hCD45+hCD3+) over time, and this was observed regardless of whether the Hu-mice had or had not received DMPA, and in those that received a high (10^5^) or low (10^3^) viral dose at challenge. This decrease in frequency of hCD45+hCD3+ cells was not seen in Hu-mice that did not become infected following intravaginal HIV-1 challenge (rows 23-34), where in some mice an increase in frequency of these cells over time was observed, likely because of an increase in reconstitution in the absence of HIV-1 infection.

# Supplemental Figures


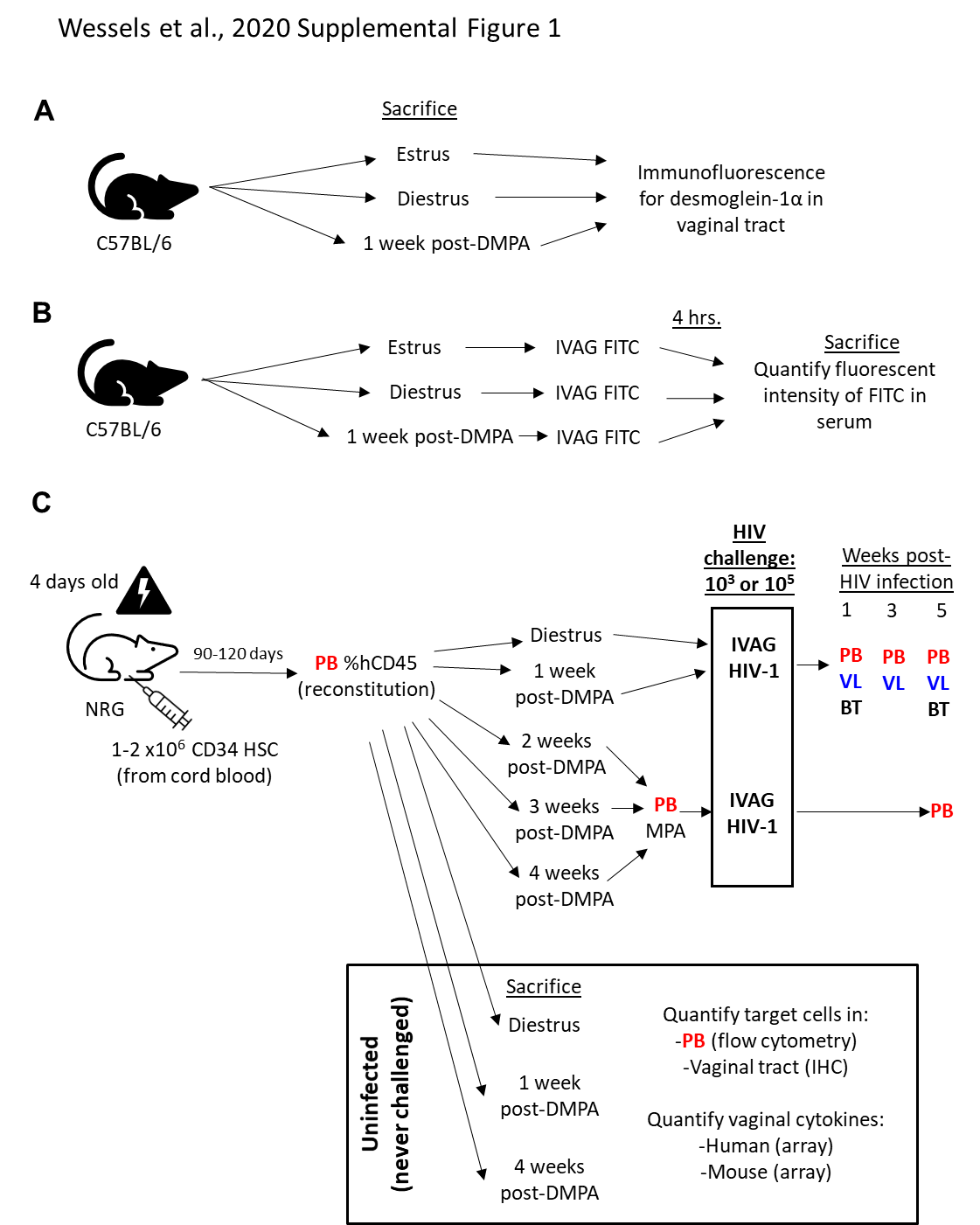


**Wessels et al., Supplemental Figure 2:**


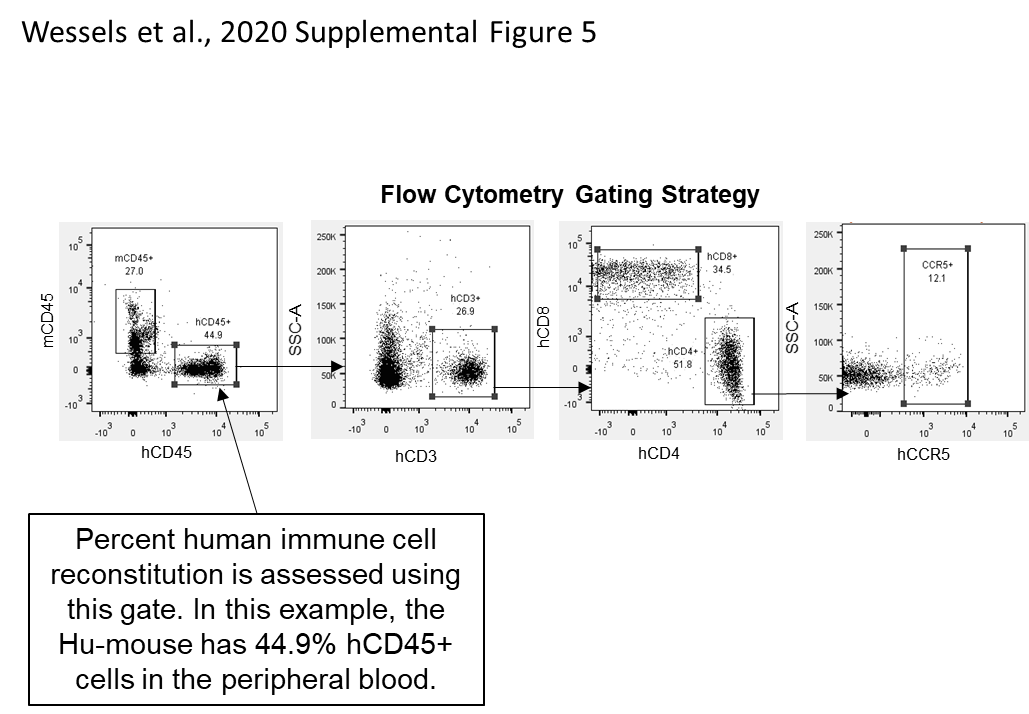


**Wessels et al., Supplemental Figure 3:**
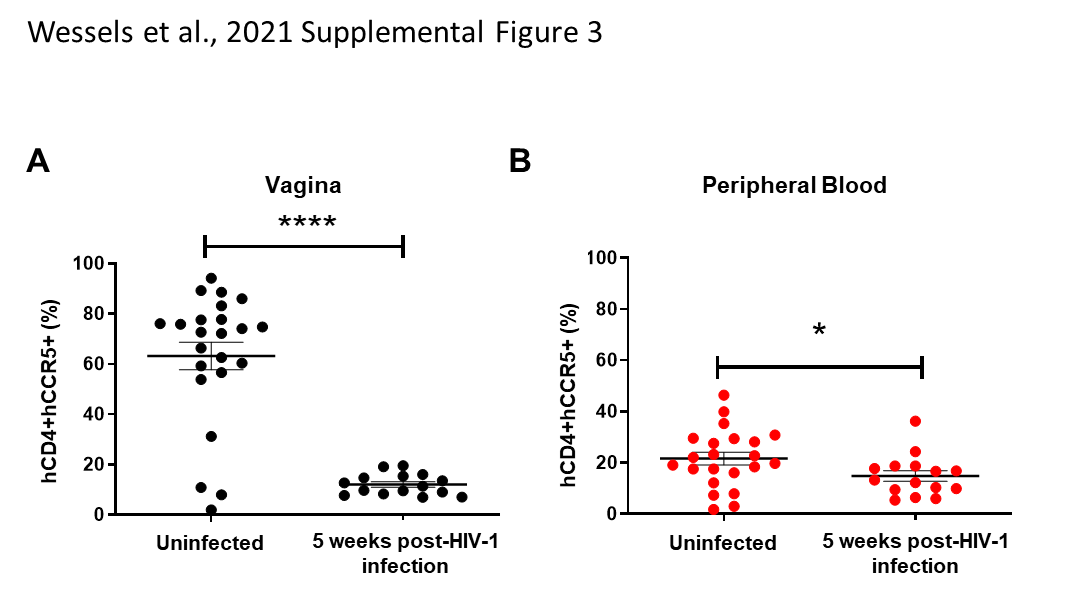


**Wessels et al., Supplemental Figure 4:**
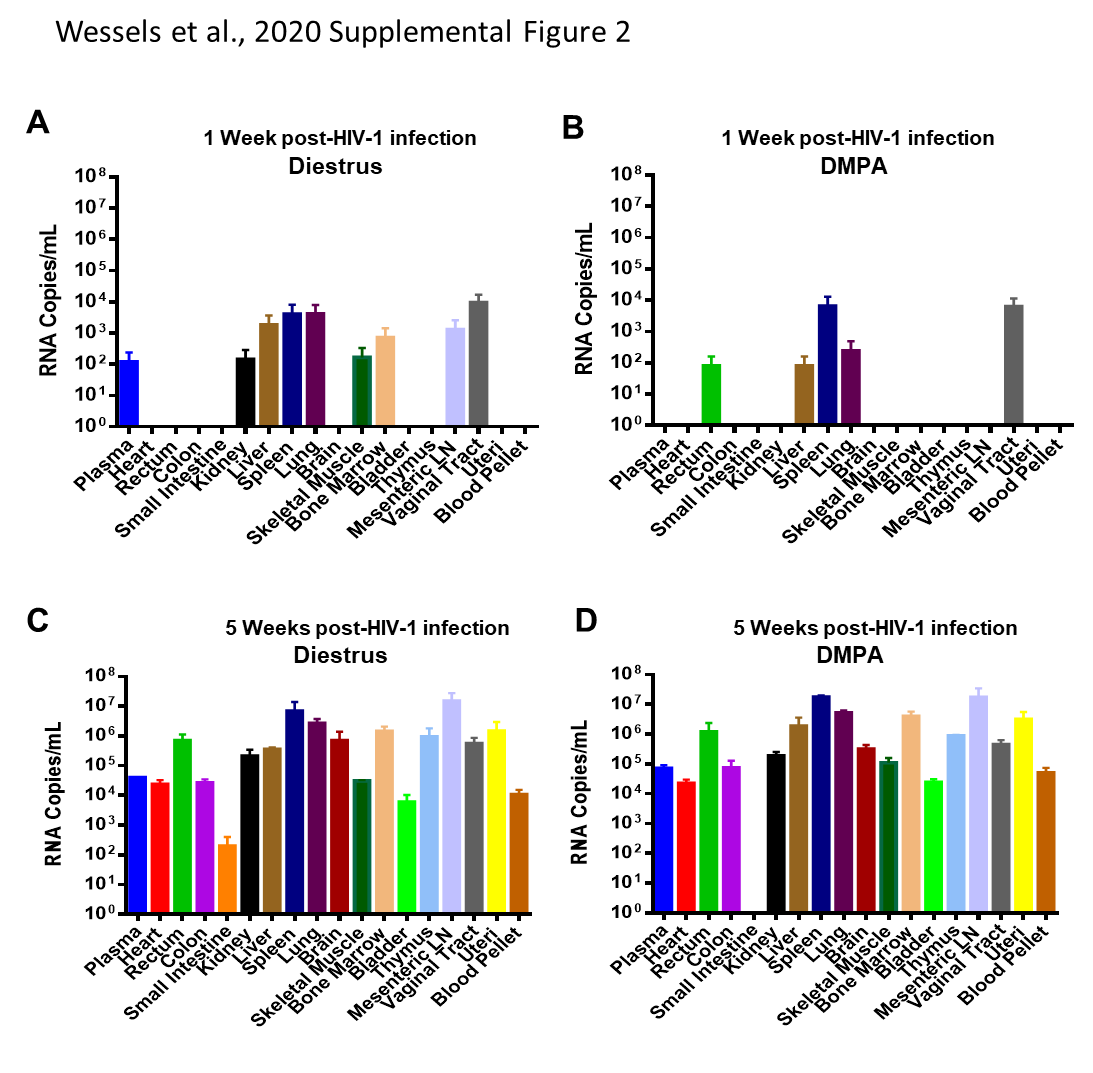


**Wessels et al., Supplemental Figure 5:**


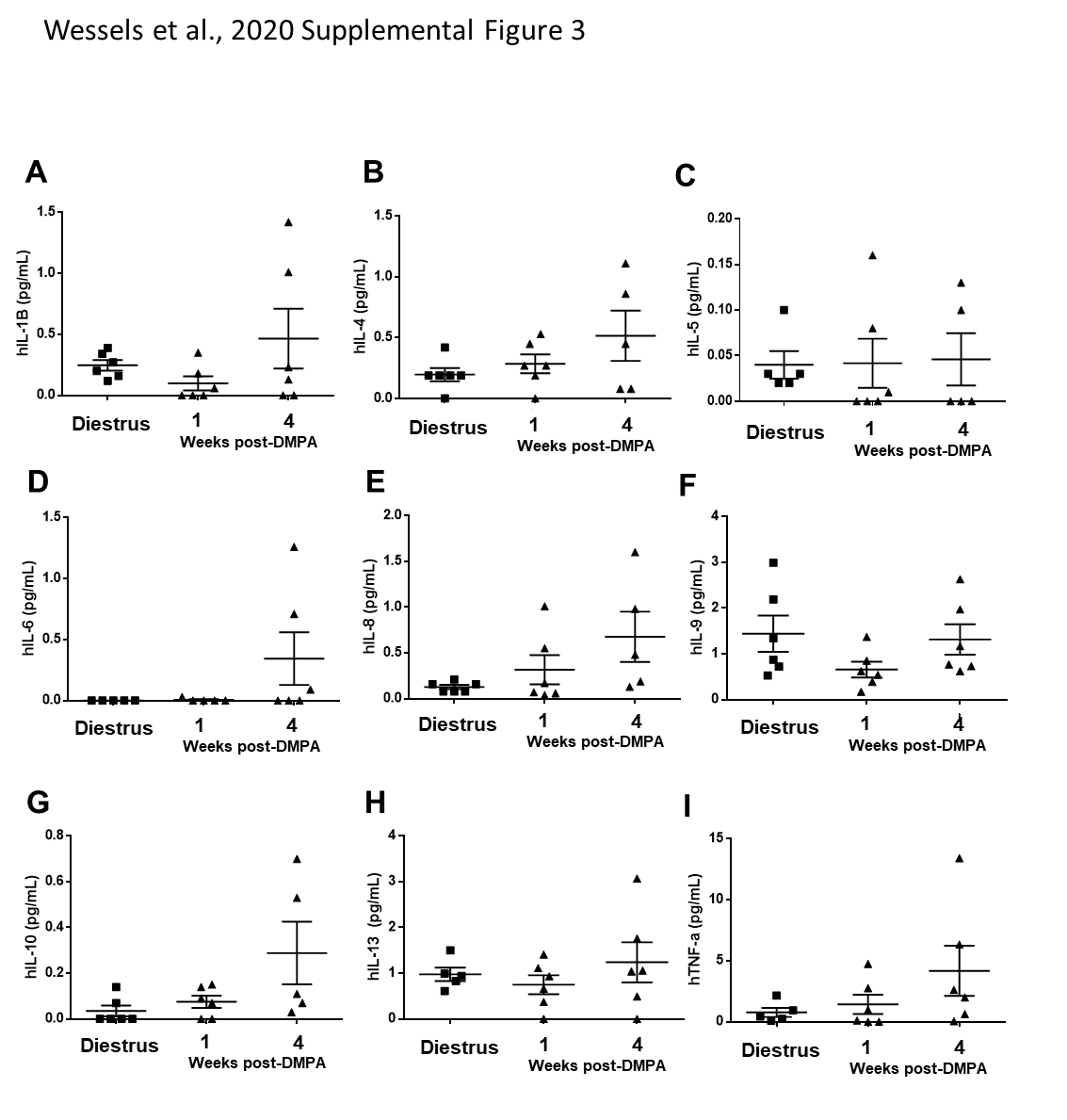


**Wessels et al., Supplemental Figure 5 (continued):**


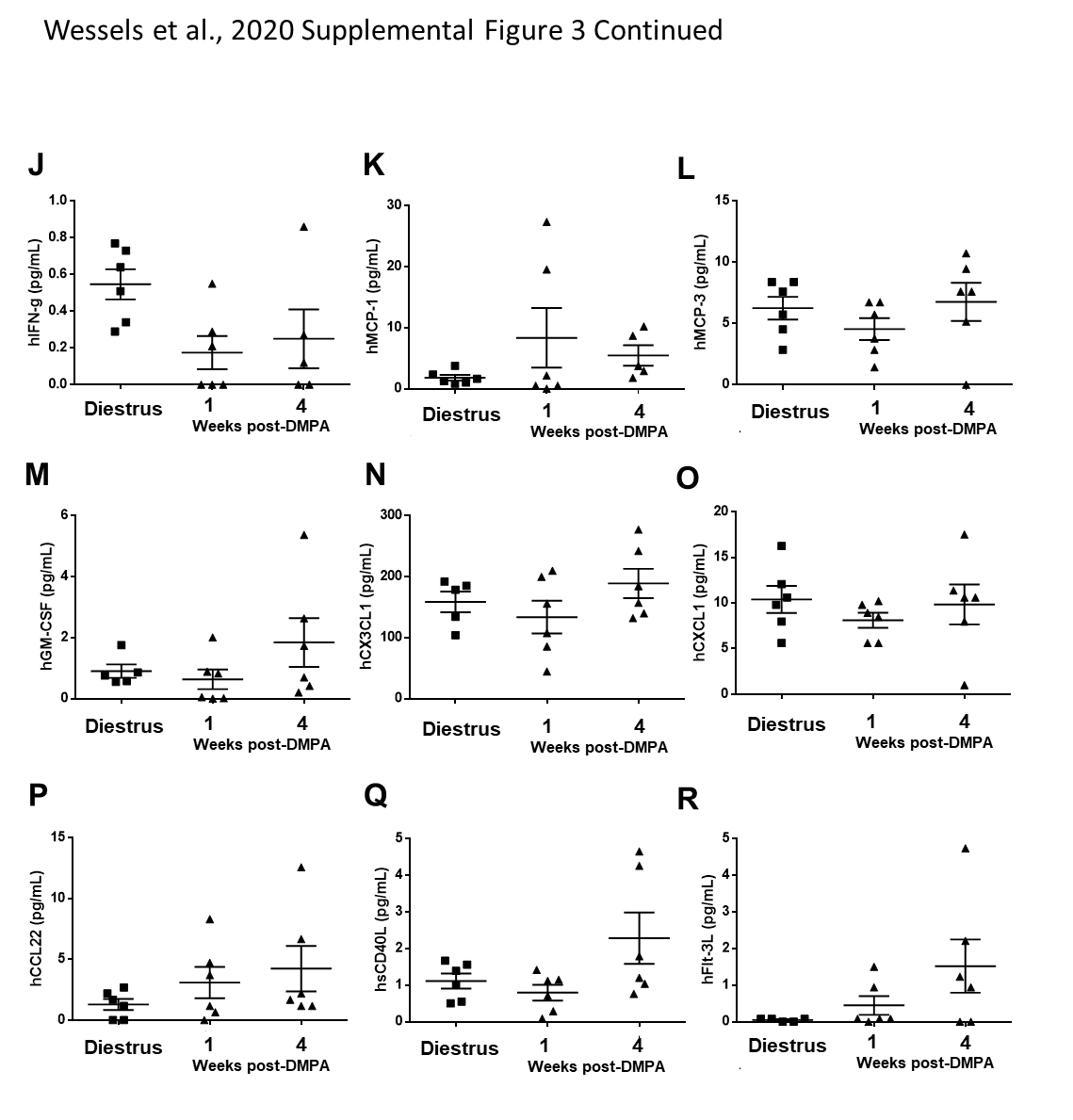


**Wessels et al., Supplemental Figure 6:**


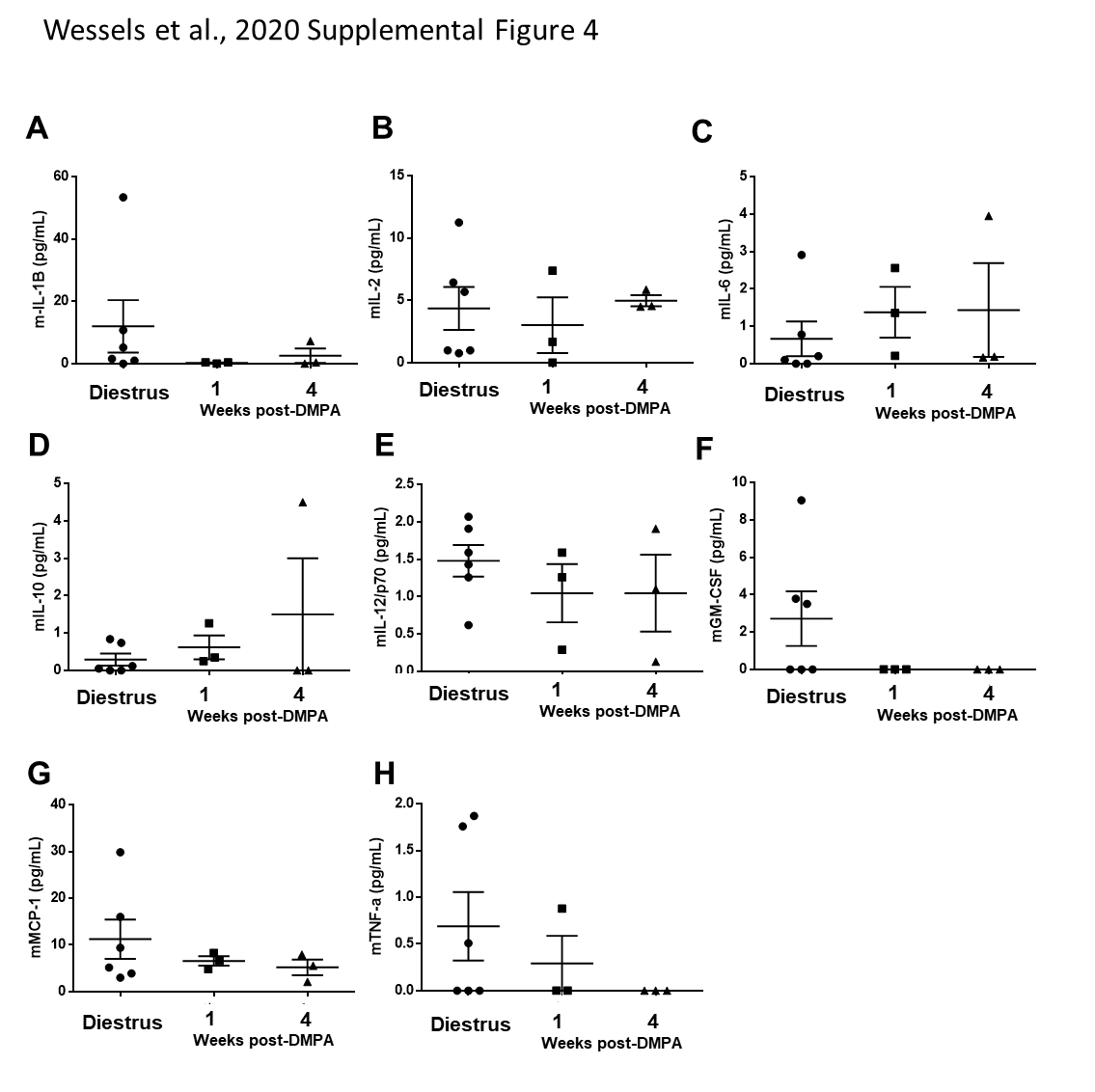

Supplement: Supplementary file 1 — Supplementary Information 1. [file 41598_2021_83242_MOESM1_ESM.doc]
